# Monoallelic *IRF5* deficiency in B cells prevents murine lupus

**DOI:** 10.1172/jci.insight.141395

**Published:** 2021-08-09

**Authors:** Alex Pellerin, Kei Yasuda, Abraham Cohen-Bucay, Vanessa Sandra, Prachi Shukla, Barry K. Horne Jr, Kerstin Nündel, Gregory A. Viglianti, Yao Xie, Ulf Klein, Ying Tan, Ramon G. Bonegio, Ian R. Rifkin

**Affiliations:** 1 Nephrology Section, Department of Medicine, Boston University School of Medicine, Boston, Massachusetts, USA.; 2 Biogen, Cambridge, Massachusetts, USA.; 3 Department of Medicine, University of Massachusetts Medical School, Worcester, Massachusetts, USA.; 4 Department of Microbiology, Boston University School of Medicine, Boston, Massachusetts, USA.; 5 Division of Haematology & Immunology, Leeds Institute of Medical Research at St. James’s, School of Medicine, University of Leeds, Leeds, United Kingdom.; 6 Nephrology Section, Department of Medicine, VA Boston Healthcare System, Boston, Massachusetts, USA.

**Keywords:** Autoimmunity, Autoimmune diseases

## Abstract

Gain-of-function polymorphisms in the transcription factor IFN regulatory factor 5 (IRF5) are associated with an increased risk of developing systemic lupus erythematosus. However, the IRF5-expressing cell type(s) responsible for lupus pathogenesis in vivo is not known. We now show that monoallelic *IRF5* deficiency in B cells markedly reduced disease in a murine lupus model. In contrast, similar reduction of IRF5 expression in macrophages, monocytes, and neutrophils did not reduce disease severity. B cell receptor and TLR7 signaling synergized to promote IRF5 phosphorylation and increase IRF5 protein expression, with these processes being independently regulated. This synergy increased B cell–intrinsic IL-6 and TNF-α production, both key requirements for germinal center (GC) responses, with IL-6 and TNF-α production in vitro and in vivo being substantially lower with loss of 1 allele of *IRF5*. Mechanistically, TLR7-dependent IRF5 nuclear translocation was reduced in B cells from *IRF5*-heterozygous mice. In addition, we show in multiple lupus models that IRF5 expression was dynamically regulated in vivo with increased expression in GC B cells compared with non-GC B cells and with further sequential increases during progression to plasmablasts and long-lived plasma cells. Overall, a critical threshold level of IRF5 in B cells was required to promote disease in murine lupus.

## Introduction

Systemic lupus erythematosus (SLE) is a chronic autoimmune disease characterized by autoantibody production, inflammation, and tissue damage in multiple organs resulting from an overactivation of the immune system through various mechanisms (1, 2). Disease development is due to a combination of genetic and environmental factors, among which polymorphisms in IFN regulatory factor 5 (*IRF5*) have been strongly associated with an increased risk of developing SLE (3, 4). Although the exact mechanism of how these polymorphisms in *IRF5* lead to an increased risk of developing SLE is incompletely understood, it is thought that they lead to increased levels of IRF5 protein expression and/or functional change (3, 4). Notably, global homozygous or heterozygous deficiency of *IRF5* has conferred protection in many mouse models of lupus (5–10). Thus, there is both human and mouse genetic evidence that suggests that IRF5 expression levels contribute to disease pathogenesis. However, the previous studies of global heterozygous and homozygous gene deficiencies (5–10) do not address how cell type–specific expression of IRF5 impacts disease and how IRF5 expression and activation are modulated in the pathogenic cell type(s) during disease development.

IRF5 is a transcription factor that plays an important role in regulating immune responses downstream of TLRs, specifically, TLR-3, TLR-4, TLR-7, and TLR-9, and other innate immune receptors, including nucleotide-binding oligomerization domain 2, retinoic acid inducible gene I, and dectin-1 (11–15). Of these, TLR7 has been most closely associated with disease pathogenesis in mouse lupus models (16). Similar to other IRF family members, IRF5 resides predominantly in the cytosol in latent form as a monomer in unstimulated cells. Upon activation, specific serine residues in the C-terminal region are phosphorylated, which causes IRF5 to homodimerize or heterodimerize and undergo nuclear translocation. The IRF5 dimer then assembles with transcriptional coactivators such as CREB-binding protein or p300 in the nucleus, which enables promoter binding and the induction of gene transcription (17, 18).

Although IRF5 is also expressed outside the immune system, it is constitutively expressed in B cells, myeloid DCs (mDCs), plasmacytoid DCs (pDCs), and monocytes/macrophages (19, 20) and plays a key role in the induction of a number of proinflammatory cytokines and chemokines (11, 21). The extent of involvement of IRF5 in cytokine production is both cell type dependent and stimulus dependent (3, 20). Beyond cytokine production, IRF5 has also been linked to promoting M1 macrophage polarization (22), IgG2a/c class-switching (6), and upregulation of costimulatory molecules and Blimp-1 in B cells (23, 24). Because B cells, mDCs, pDCs, and monocytes/macrophages have all been implicated in lupus pathogenesis, it is plausible that IRF5 in any 1 or more of these cell types may be required for disease pathogenesis.

B lineage cells are thought to be central to the pathogenesis of lupus, although the optimal therapeutic intervention targeting this lineage is not yet established (25). Clinical trials of B cell depletion in lupus have shown variable efficacy, leading to a shift in focus to developing more effective B cell–depleting strategies and also targeting long-lived plasma cell populations (26). In mouse models of lupus, B cell deficiency is protective (16). A role for B cell–intrinsic TLR signaling was demonstrated in bone marrow chimera studies, showing that B cell–specific expression of the TLR adaptor protein MyD88 is required for disease development (27). Given that B cells and their expression of key molecules in the TLR signaling pathways are important in disease development, we hypothesized that the function of IRF5 in B cells might be a central component of pathogenesis. Furthermore, the fact that heterozygous global deletion of *IRF5* markedly reduces disease in lupus models suggested that loss of 1 allele of *IRF5* in B cells might confer protection (5, 10, 28, 29).

To examine the role of B cell–specific IRF5, we conditionally deleted 1 allele of *IRF5* in the *FcγRIIB*^−/−^*Yaa* mouse lupus model, the model we previously used to demonstrate the effect of global homozygous and heterozygous *IRF5* deletion (5). We found that heterozygous deletion of *IRF5* in B cells led to a marked reduction in disease severity. In contrast, a similar reduction of IRF5 expression in macrophages, monocytes, and neutrophils did not impact disease. Thus, there was a critical threshold level of IRF5 expression in B cells that was necessary to induce disease, and this suggests that future therapeutic approaches targeting IRF5 in SLE will need to effectively reduce IRF5 expression or activity in B cells.

## Results

### IRF5 expression in bone marrow–derived cells is required for the development of autoimmune disease.

IRF5 is expressed in both immune cells and non–bone marrow–derived cells (19), and previous reports have suggested a role for IRF5 in non–bone marrow–derived cells in lupus pathogenesis (30). To evaluate the relative contribution of IRF5 expression in bone marrow–derived cells to disease pathogenesis in the *FcγRIIB*^−/−^*Yaa* model, we performed bone marrow chimera studies. Bone marrow from *FcγRIIB*^−/−^ *Yaa* *IRF5*^+/+^ or *FcγRIIB*^−/−^ *Yaa* *IRF5*^–/–^ mice was transferred into irradiated C57BL/6 (B6) recipients. Disease parameters were evaluated 28 weeks after transfer. Mice that received the *FcγRIIB*^−/−^ *Yaa* *IRF5*^+/+^ bone marrow developed splenomegaly, antinuclear autoantibodies (ANAs), and kidney disease, whereas mice that received the *FcγRIIB*^−/−^ *Yaa* *IRF5*^–/–^ bone marrow did not. These studies demonstrate that IRF5 expression in bone marrow–derived cells was sufficient to drive the lupus-like disease observed in *FcγRIIB*^−/−^*Yaa* mice (Supplemental Figure 1; supplemental material available online with this article; https://doi.org/10.1172/jci.insight.141395DS1).

### Generation of FcγRIIB^−/−^Yaa mice with a B cell–specific deletion of IRF5.

To study the role of IRF5 expression specifically in B cells, we first generated an *IRF5*-floxed allele on a C57BL/6 genetic background by introducing loxP sites flanking exons 3–6 including a neo cassette flanked by Frt sites to the 5*′* end of exon 3 (Supplemental Figure 2). Mice were then bred to the Flpe deleter mice to remove the neo cassette. The resulting *IRF5*^fl/fl^ mice were intercrossed with *Mb1*^cre/wt^ and *FcγRIIB*^−/−^*Yaa* mice, generating *FcγRIIB*^−/−^*Yaa* mice with a heterozygous deletion of *IRF5* in B cells (*Mb1*^cre/wt^ *IRF5*^F/+^ mice; termed *IRF5*^Δ^B) and littermate control *FcγRIIB*^−/−^*Yaa* mice with no deletion of *IRF5* in B cells (*Mb1*^wt/wt^ *IRF5*^F/+^ mice; termed *IRF5*^F/+^). We chose to focus on the heterozygous deletion of *IRF5* in B cells given our previous finding that global heterozygous *IRF5* deletion protected mice from disease to a similar extent as homozygous deletion, a finding that has been confirmed in other mouse lupus models (5, 10, 28, 29).

In Mb1cre-expressing animals, the cre transgene is driven by the CD79α promoter and results in the deletion of the floxed allele in 95% of B cells (31). In some cases, the Mb1cre transgene has been reported to induce ectopic, germ line deletion of floxed genes (32). Therefore, it was important to ensure that the deletion mediated by Mb1cre in our system was restricted to the B cell lineage.

**Figure 1:**
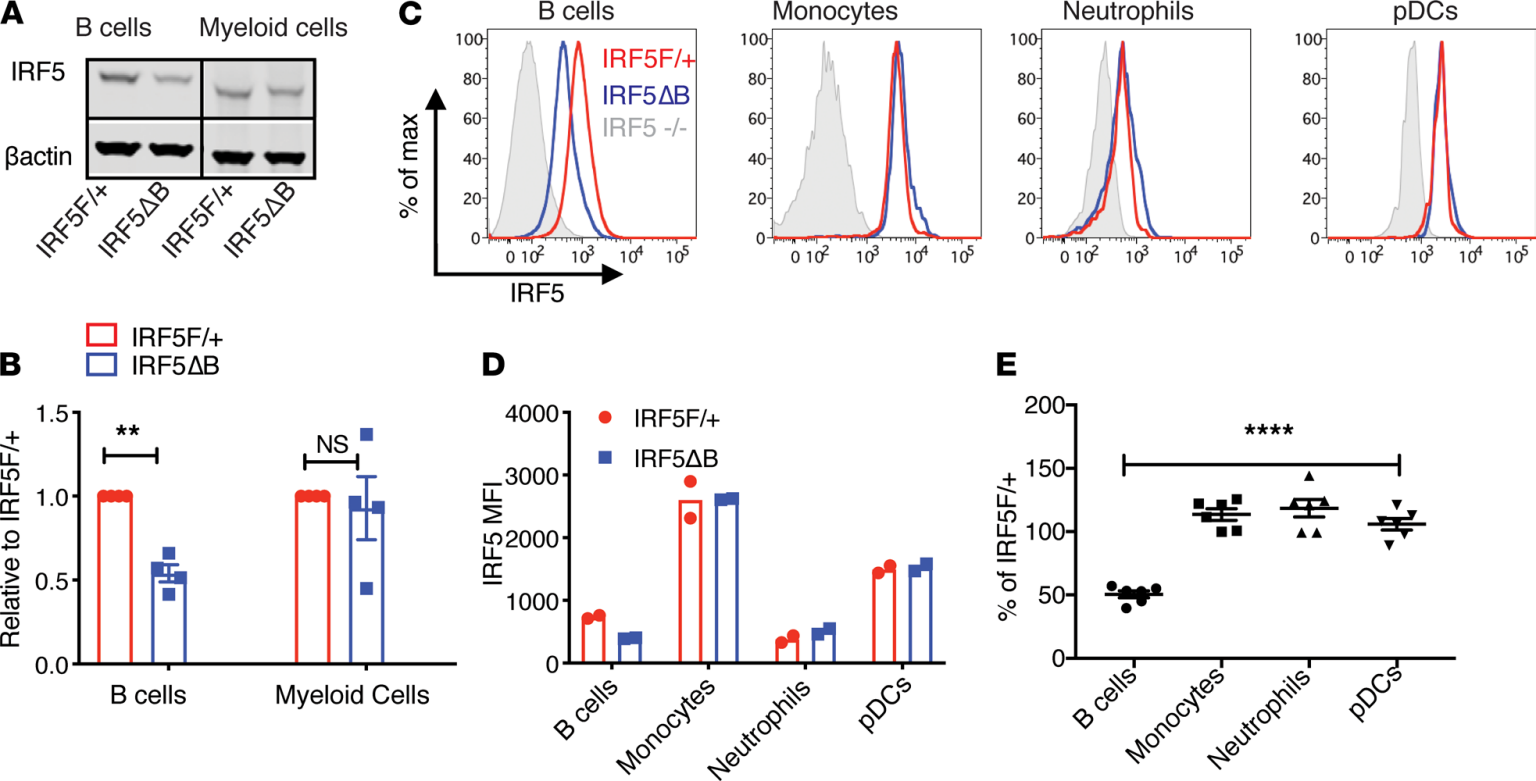
B cell–specific reduction of IRF5 expression in *IRF5^Δ^*B mice. All analyses were done in 8- to 10-week-old *FcγRIIB*^−/−^*Yaa* mice. (**A**) Representative Western blot of IRF5 protein expression in sorted splenic B cells (CD19^+^) and myeloid cells (CD11b^+^Ly6G^–^) from *IRF5*^F/+^ and *IRF5*^Δ^B mice. All lanes were run on the same gel but were noncontiguous. (**B**) Expression of IRF5 in B cells and myeloid cells from *IRF5*^Δ^B mice normalized to *IRF5*^F/+^ (*n* = 4). Data were analyzed using 2-tailed, unpaired Welch’s *t* test; **P < 0.01. (**C**) Representative flow cytometry plots of intracellular IRF5 expression in CD19^+^ B cells, CD11b^+^Ly6C^+^ monocytes, CD11b^+^Ly6G^+^ neutrophils, and CD11b^–^PDCA1^+^Ly6C^+^ pDCs from *IRF5*^F/+^, *IRF5*ΔB, and *IRF5*^–/–^ global knockout mice. (**D**) MFI values of IRF5 in B cells, monocytes, neutrophils, and pDCs from *IRF5*^F/+^ and *IRF5*^Δ^B mice (representative experiment of 3 individual experiments, *n* = 2 for each genotype). (**E**) IRF5 expression in B cells, monocytes, neutrophils, and pDCs from *IRF5*^Δ^B mice normalized to the *IRF5*^F/+^ littermate control in each experiment (*n* = 6). Data are shown as mean ± SEM and were analyzed using 1-way ANOVA with Tukey’s post hoc test; ****P < 0.0001. IRF5, IFN regulatory factor 5; pDCs, plasmacytoid DCs.

To evaluate *IRF5* deletion, we first used flow cytometry to sort mature B cells (B220^+^CD93^–^) and myeloid cells (B220^–^CD3^–^CD11b^+^Ly6G^–^) from *IRF5*^F/+^ mice expressing or not expressing Mb1cre and performed Western blot on the B cell and myeloid cell populations. Mb1cre-mediated deletion of *IRF5*^F/+^ mice resulted in an approximately 50% reduction in IRF5 expression in B cells (Figure 1, A and B). This is consistent with the reduction we observed in the B cells of *IRF5* germ line heterozygous mice (Supplemental Figure 3, A and B). Importantly there was no difference in IRF5 protein expression in myeloid cells from the *IRF5*^Δ^B and *IRF5*^F/+^ mice. To corroborate the Western blot studies and extend the analysis to specific IRF5-expressing immune cell types not captured in the myeloid gating strategy, we measured IRF5 expression by intracellular flow cytometry (Figure 1, C–E). This confirmed that IRF5 reduction in *IRF5*^F/+^ *FcγRIIB*^−/−^*Yaa* mice expressing Mb1cre was restricted to B cells as the MFI of IRF5 was reduced by approximately 50% in B cells but was not reduced in monocytes, neutrophils, or pDCs (Figure 1, C–E). The gating strategy used to define each immune cell population is shown in Supplemental Figure 3C. Figure 1D represents MFI values from 2 separate mice of each genotype from 1 experiment. Figure 1E is a compilation of MFI values from 6 *IRF5*^Δ^B mice normalized to the *IRF5*^F/+^ littermate controls from the same experiment. The intracellular staining for IRF5 was specific, because cells from *IRF5* germ line knockout mice stained with the anti-IRF5 antibody had a similar staining profile as the isotype control antibody (Supplemental Figure 3D). Taken together, these data indicate that we successfully generated *FcγRIIB*^−/−^*Yaa* mice with B cell–specific deletion of 1 allele of *IRF5*.

### Monoallelic deletion of IRF5 in B cells reduces splenomegaly and T cell activation in FcγRIIB^−/−^Yaa mice.

**Figure 2:**
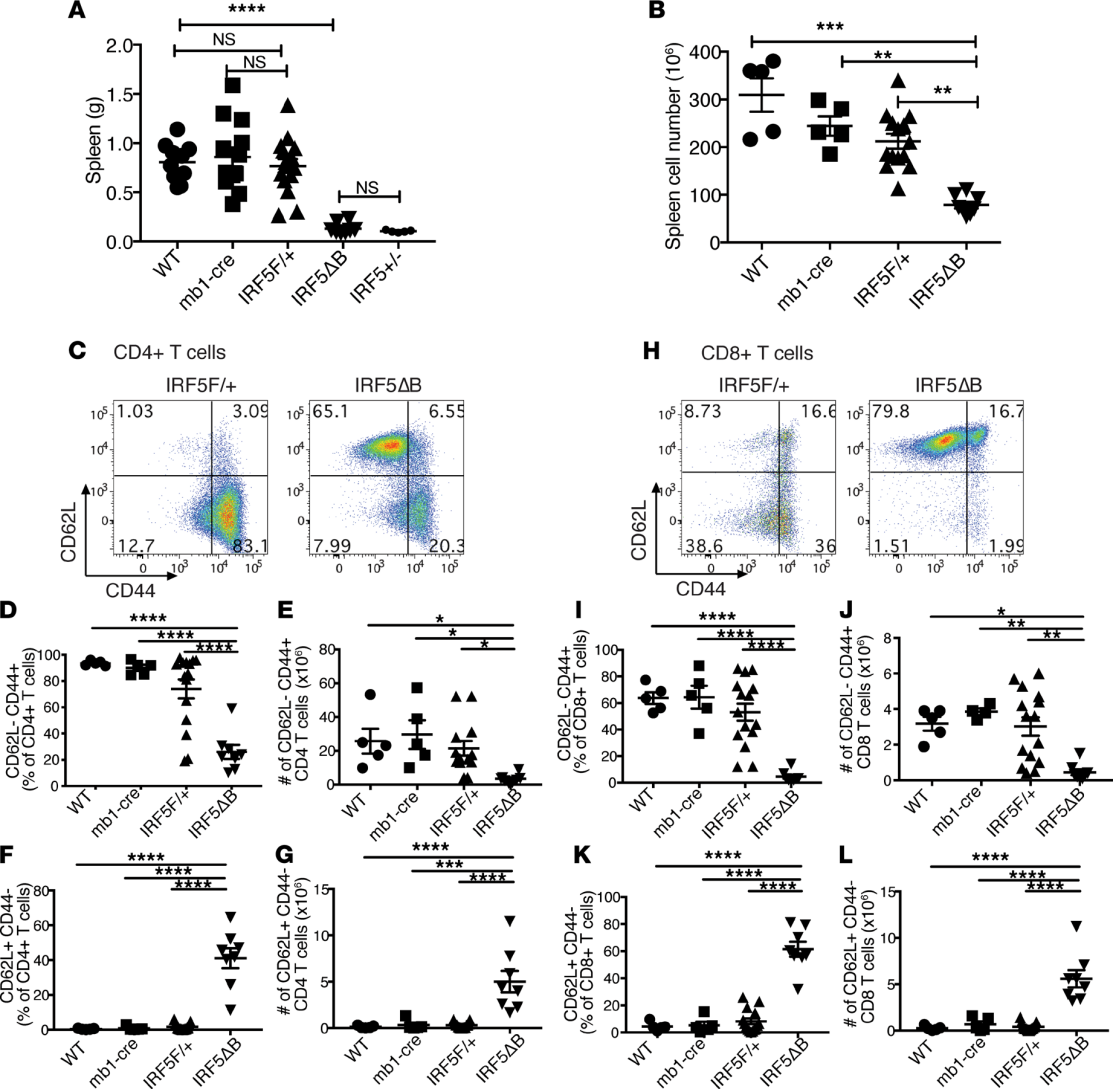
Splenomegaly and T cell activation are reduced in *IRF5^Δ^*B mice. All analyses were done in 5-month-old *FcγRIIB*^−/−^*Yaa* mice. (**A**) Spleen weights from WT (*n* = 10), *mb1cre* (*n* = 13), *IRF5*^F/+^ (*n* = 16), *IRF5*^Δ^B (*n* = 8), and *IRF5*^+/–^ (global heterozygous deletion, *n* = 5) mice. (**B**) Splenic cell counts from WT (*n* = 5), *mb1cre* (*n* = 5), *IRF5*^F/+^ (*n* = 14), and *IRF5*^Δ^B (*n* = 8) mice. (**C**) Representative flow cytometry plots of CD4^+^CD62L^–^CD44^+^ (effector/memory) and CD62L^+^CD44^–^ (naive) T cells from spleen of *IRF5*^F/+^ and *IRF5*^Δ^B mice. (**D** and **E**) Percentage and number of CD62L^–^CD44^+^ CD4^+^ T cells from WT (*n* = 5), *mb1cre* (*n* = 5), *IRF5*^F/+^ (*n* = 15), and *IRF5*^Δ^B (*n* = 8) mice. (**F** and **G**) Percentage and number of CD62L^+^CD44^–^CD4^+^ T cells. (**H**) Representative flow cytometry plots of CD8^+^CD62L^–^CD44^+^ (effector/memory) and CD62L^+^CD44^–^ (naive) T cells from spleen of *IRF5*^F/+^ and *IRF5*^Δ^B mice. (**I** and **J**) Percentage and number of CD62L^–^CD44^+^CD8^+^ T cells from WT (*n* = 5), *mb1cre* (*n* = 5), *IRF5*^F/+^ (*n* = 15), and *IRF5*^Δ^B (*n* = 8) mice. (**K** and **L**) Percentage and number of CD62L^+^CD44^–^CD8^+^ T cells. Data are shown as mean ± SEM and were analyzed using 1-way ANOVA with Tukey’s post hoc test; *P < 0.05, **P < 0.01, ***P < 0.001, ****P < 0.0001. IRF5, IFN regulatory factor 5.

Splenomegaly is seen in 10%–45% of patients with SLE and in multiple mouse models of lupus-like disease (33). Therefore, we evaluated the effect of heterozygous deletion of *IRF5* in B cells on splenomegaly and found that it was markedly reduced and to the same extent as that seen in global *IRF5*-heterozygous *FcγRIIB*^−/−^*Yaa* mice (Figure 2A). No reduction in splenomegaly was seen in *FcγRIIB*^−/−^*Yaa* mice expressing Mb1cre alone or the *IRF5*-floxed allele alone. The reduction in spleen weight also correlated with a reduction in overall spleen cell number (Figure 2B). There was no significant difference in the number of CD3^+^ T cells and CD19^+^ B cells between the genotypes, indicating that the splenomegaly in this model was due to the expansion of a non–B cell, non–T cell population (Supplemental Figure 4).

We next evaluated the effect of heterozygous deletion of *IRF5* in B cells on T cell activation using flow cytometry, to assess T cell activation based on CD44 and CD62L expression. In both the CD4^+^ (Figure 2, C–E) and CD8^+^ (Figure 2, H–J) T cell populations, we found a dramatic reduction in the percentage and number of effector/memory (CD62L^–^ CD44^+^) T cells in the *IRF5*^Δ^B mice compared with the *FcγRIIB*^−/−^*Yaa* mice and the littermate *FcγRIIB*^−/−^*Yaa* mice expressing Mb1cre alone or the *IRF5*-floxed allele alone. Notably, as with splenomegaly, no impact on T cell activation was seen in *FcγRIIB*^−/−^*Yaa* mice expressing Mb1cre alone or the *IRF5*-floxed allele alone compared with *FcγRIIB*^−/−^*Yaa* mice. Conversely, the percentage and number of naive (CD62L^+^CD44^–^) T cells in the *IRF5*^Δ^B mice was greatly increased compared with the other experimental groups for both the CD4^+^ (Figure 2, F and G) and CD8^+^ (Figure 2, K and L) populations. These data indicate that heterozygous deletion of *IRF5* in B cells markedly reduced splenomegaly and prevented T cell activation in *FcγRIIB*^−/−^*Yaa* mice and that the *Mb1cre* allele or the *IRF5*-floxed allele alone had no impact on these measurements.

### Monoallelic deletion of IRF5 in B cells reduces serum autoantibodies, splenic plasmablasts, and bone marrow plasma cells.

**Figure 3:**
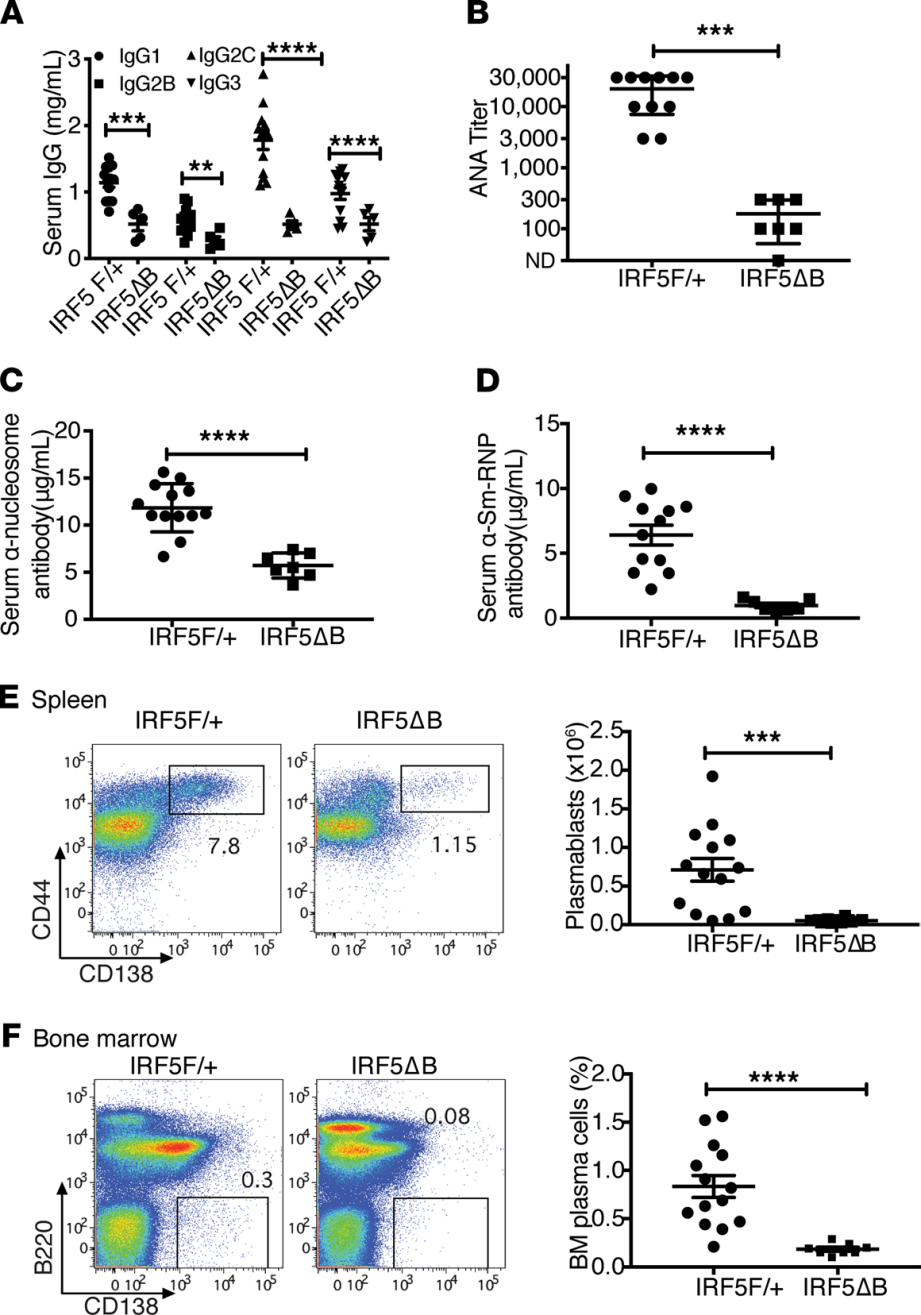
Serum IgG, autoantibodies, and plasma cells are reduced in *IRF5^Δ^*B mice. All analyses were done in 5-month-old *FcγRIIB*^−/−^*Yaa* mice. (**A**–**D**) *IRF5*^F/+^ (*n* = 11) and *IRF5*^Δ^B mice (*n* = 5–7) were analyzed. (**A**) Serum IgG isotype concentrations. (**B**) Serum antinuclear autoantibody titers. (**C**) Serum anti-nucleosome IgG concentration. (**D**) Serum anti-Sm/RNP IgG concentrations. (**E**) Representative flow cytometry plots and total numbers of splenic plasmablasts in *IRF5*^F/+^ (*n* = 15) and *IRF5*^Δ^B (*n* = 8) mice. (**F**) Representative flow cytometry plots and percentages of bone marrow plasma cells in *IRF5*^F/+^ (*n* = 15) and *IRF5*^Δ^B (*n* = 8) mice. Data are shown as mean ± SEM and were analyzed using 2-tailed, unpaired Welch’s *t* test; **P < 0.01, ***P < 0.001, ****P < 0.0001. IRF5, IFN regulatory factor 5.

Antinuclear autoantibodies are a hallmark of SLE and are thought to contribute to disease pathogenesis (34). We first evaluated the effect of B cell–specific heterozygous deletion of *IRF5* on serum IgG isotype levels and found that this significantly reduced the levels of all 4 IgG isotypes, IgG1, IgG2b, IgG2c, and IgG3 (Figure 3A). We next measured serum ANA and, as expected, found that *IRF5*^F/+^ mice had a high ANA titer as measured by immunofluorescence on HEp2 cells (Figure 3B). However, there was a marked reduction of ANA titer in the *IRF5*^Δ^B mice (Figure 3B). This reduction was seen in both autoantibodies directed against DNA-containing autoantigens (antinucleosome; Figure 3C) and autoantibodies directed against RNA-containing autoantigens (anti-Sm/RNP; Figure 3D).

To determine how B cell–specific heterozygous deletion of *IRF5* might cause the observed reduction in serum IgG and autoantibody levels, we evaluated plasmablast numbers in the spleen and plasma cell percentages in the bone marrow. It has been recently reported that B cell–intrinsic IRF5 expression is necessary for human B cell differentiation into plasmablasts in vitro (35), and *IRF5*-deficient mice have fewer plasma cells (23). We found that there was an appreciable population of plasmablasts in the spleens of *IRF5*^F/+^ mice at 5 months of age and that this plasmablast population was greatly reduced in the *IRF5*^Δ^B mice (Figure 3E). Similarly, there was a large reduction in plasma cells in the bone marrow compartment of the *IRF5*^Δ^B mice at 5 months of age compared with the *IRF5*^F/+^ mice at 5 months of age (Figure 3F). These data explain the reduction of IgG and autoantibodies observed with the heterozygous deletion of *IRF5* in B cells in the *FcγRIIB*^−/−^*Yaa* mice and demonstrate that B cell–intrinsic IRF5 was necessary for plasmablast and plasma cell development in a mouse model of lupus.

### Monoallelic deletion of IRF5 in B cells reduces disease manifestations in the kidney.

**Figure 4:**
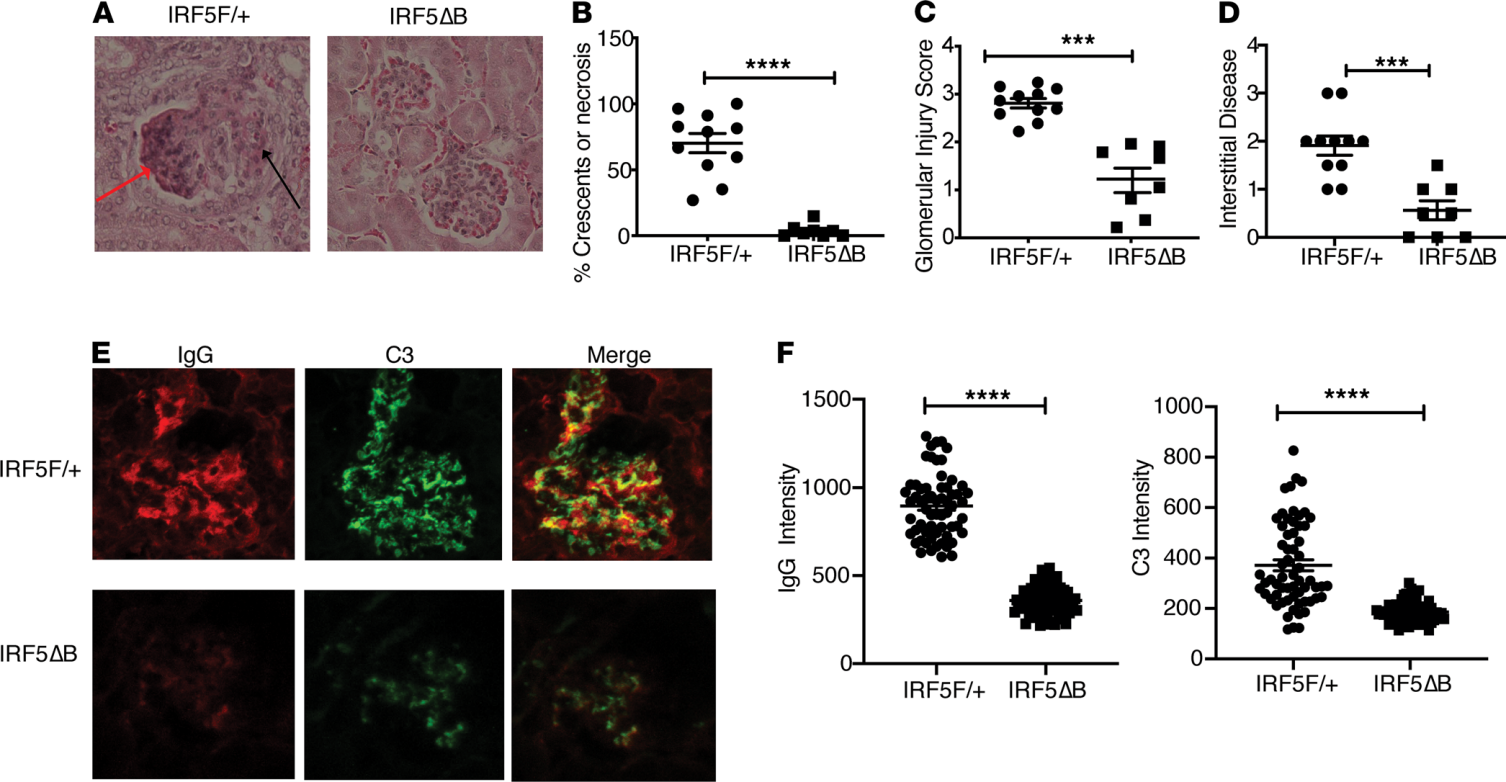
Decreased renal disease in *IRF5^Δ^*B mice. All analyses were done in 5-month-old *FcγRIIB*^−/−^*Yaa* mice. (**A**) Representative renal histology of *IRF5*^F/+^ and *IRF5*^Δ^B mice. Red arrow depicts necrotic cells; black arrow depicts cellular crescent (original magnification, ×20). (**B**–**D**) Quantification of renal disease by (**B**) percentage of glomeruli with crescents or necrosis, (**C**) glomerular injury score, and (**D**) interstitial disease. *IRF5*^F/+^ (*n* = 11) and *IRF5*B (*n* = 8). (**E**) Representative examples and (**F**) quantitation of glomerular IgG and complement C3 deposition measured by fluorescence intensity in 11–14 glomeruli per mouse from 5 mice per group. All scored glomeruli are shown (original magnification, ×20). Data are shown as mean ± SEM and were analyzed using 2-tailed, unpaired Welch’s *t* test; ***P < 0.001, ****P < 0.0001. IRF5, IFN regulatory factor 5.

The renal disease in lupus patients and in many mouse lupus models is characterized by immune complex deposition, complement activation, and glomerular inflammation with resultant glomerular crescent formation and cellular injury, often accompanied by tubulointerstitial disease (36). To assess whether the deletion of 1 allele of *IRF5* in B cells might impact renal disease, we measured glomerular inflammation and injury as well as interstitial disease in kidneys from 4- to 5-month-old *IRF5*^F/+^ and *IRF5*^Δ^B mice. The *IRF5*^F/+^ mice all had glomerular crescents and/or necrosis, whereas these features were essentially absent in the *IRF5*B mice (Figure 4, A and B). The more severe renal disease in the *IRF5*^F/+^ mice was also evident as assessed by the overall glomerular injury score and interstitial disease (Figure 4, C and D) and by the extent of immune complex and complement deposition (Figure 4, E and F).

### The deletion of IRF5 in macrophages, monocytes, and neutrophils did not reduce disease severity in FcγRIIB^−/−^Yaa mice.

**Figure 5:**
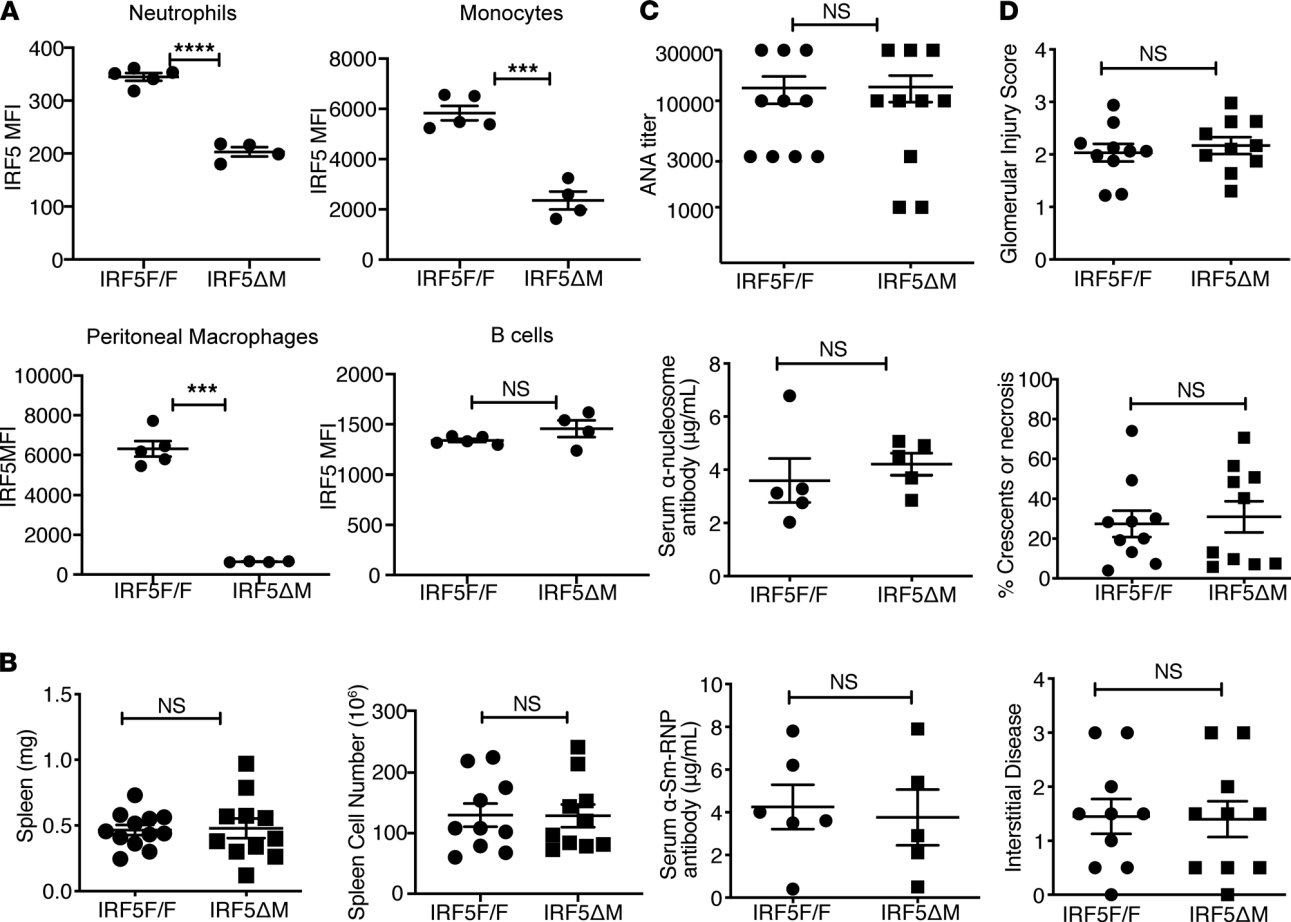
LysMcre-mediated deletion of *IRF5* does not reduce disease in *FcγRIIB^−/−^Yaa* mice. All analyses were done in *FcγRIIB*^−/−^*Yaa* littermates that either did not express LysMcre (*IRF5*^fl/fl^) or did express LysMcre (*IRF5*^Δ^M). (**A**) Flow cytometry quantitation of intracellular IRF5 expression (MFI values) in neutrophils (CD11b^+^Ly6G^+^), monocytes (CD11b^+^Ly6C^+^), and B cells (CD19^+^) from spleen, and in peritoneal macrophages (F/480^+^CD11b^+^), in 8- to 10-week-old *FcγRIIB*^−/−^*Yaa* littermates (*n* = 4 or *n* = 5). Data are shown as mean ± SEM and were analyzed using 2-tailed, unpaired Welch’s *t* test; ***P < 0.001, ****P < 0.0001. (**B**–**D**) All analyses were done in 5-month-old *FcγRIIB*^−/−^*Yaa* littermates. (**B**) Spleen weight and cell counts from *IRF5*^fl/fl^ (*n* = 10–12) and *IRF5*^Δ^M mice (*n* = 10–12). (**C**) Serum antinuclear autoantibody titers, serum anti-nucleosome IgG concentration, and serum anti-Sm/RNP IgG concentration (*n* = 5–10). (**D**) Quantification of renal disease by glomerular injury score, percentage of glomeruli with crescents or necrosis, and interstitial disease (*n* = 10). Data are shown as mean ± SEM and were analyzed using 2-tailed, unpaired Welch’s *t* test. IRF5, IFN regulatory factor 5.

Although our data demonstrated that IRF5 expression in B cells was required for disease pathogenesis, this did not exclude the possibility that IRF5 expression in other bone marrow–derived cells might also be required. To address this possibility, we deleted *IRF5* in macrophages, monocytes, and neutrophils in the *FcγRIIB*^−/−^*Yaa* model using the LysMcre system (37, 38). We found substantial reductions in IRF5 expression in peritoneal macrophages and splenic monocytes and neutrophils in the *IRF5*^F/F^ *FcγRIIB*^−/−^*Yaa* mice expressing LysMcre (termed *IRF5*^Δ^M) compared with littermates not expressing LysMcre (termed *IRF5*^fl/fl^), but we found no difference in IRF5 expression in B cells (Figure 5A). However, we found no difference in spleen size, spleen cell number, autoantibody levels, or renal disease between the *IRF5*^fl/fl^ mice and their *IRF5*^Δ^M littermates at 5 months of age (Figure 5, B–D).

### Critical threshold level of IRF5 in B cells is necessary for the formation of spontaneous germinal center B cells, T follicular helper cells, and age-associated B cells in FcγRIIB^−/−^Yaa mice early in disease pathogenesis.

Our data show that the *IRF5*^Δ^B mice were protected from the development of lupus disease manifestations. To better understand mechanistically how IRF5 expression in B cells was contributing to this phenotype, we evaluated mice at 8–10 weeks of age prior to the appearance of overt signs of disease.

**Figure 6:**
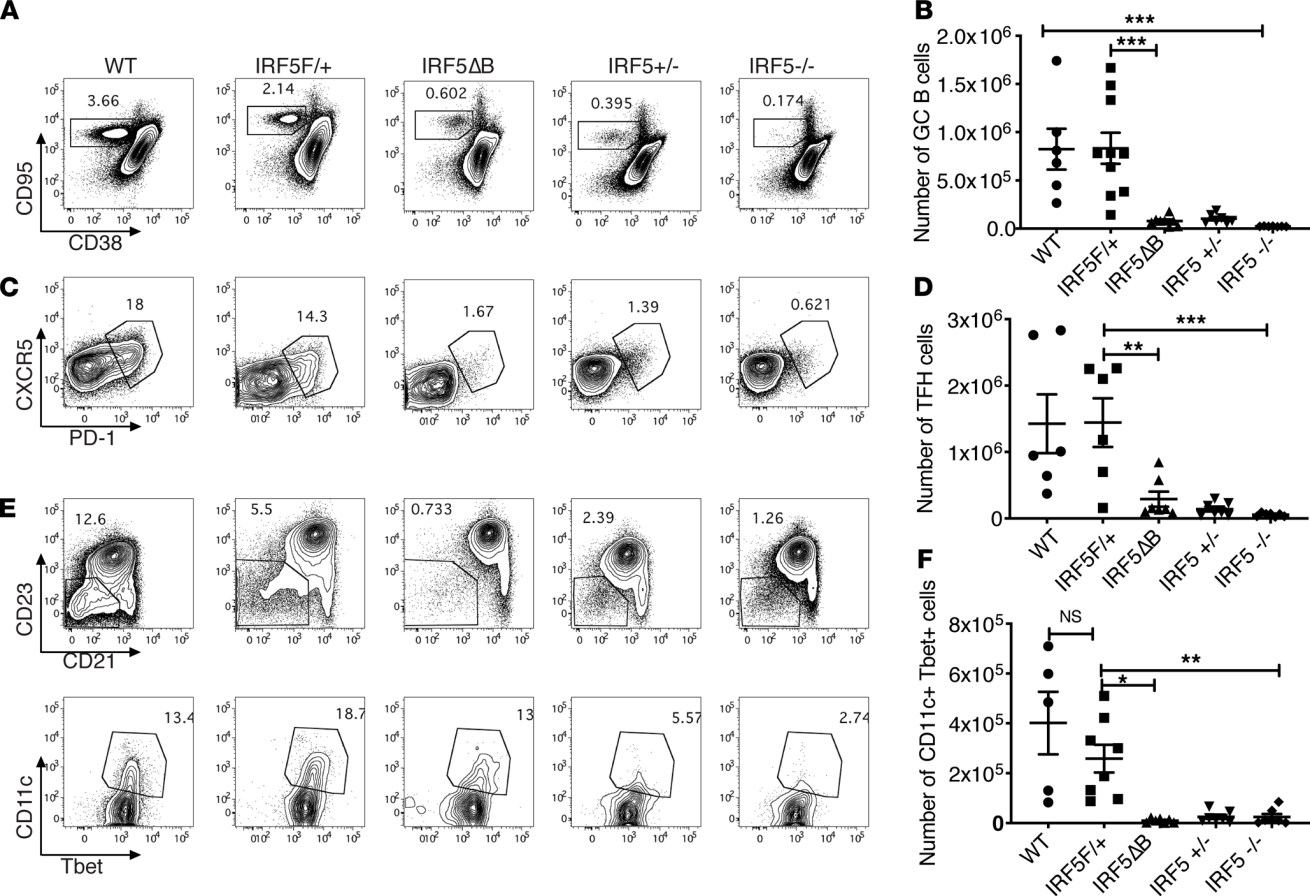
GC B cells, Tfhs, and T-bet^+^ B cells are reduced in the spleens of *IRF5^Δ^*B mice. Spleen cells from 8- to 10-week-old *FcγRIIB*^−/−^*Yaa* WT (*n* = 6), *IRF5*^F/+^ (*n* = 10), *IRF5*^Δ^B (*n* = 8), *IRF5*^+/–^ (global heterozygous deletion, *n* = 7), and *IRF5*^–/–^ (global homozygous deletion, *n* = 7) mice were analyzed. (**A** and **B**) Representative flow cytometry plots and total numbers of CD95^+^CD38^–^ GC B cells (gated on CD19^+^B220^+^ cells). (**C** and **D**) Representative flow cytometry plots and total numbers of CXCR5^+^PD-1^+^ Tfhs (gated on CD3^+^CD4^+^). (**E**) Upper panel indicates CD23^–^CD21^–^ B cells (gated on B220^+^CD19^+^CD43^–^CD93 cells); lower panel indicates T-bet^+^CD11c^+^ ABCs gated on the CD23^–^CD21^–^ B cells shown in the upper panel. A representative example is shown. (**F**) Total number of T-bet^+^CD11c^+^ ABCs. Data are shown as mean ± SEM and were analyzed using 1-way ANOVA with Tukey’s post hoc test; *P < 0.05, **P < 0.01, ***P < 0.001. GC, germinal center; Tfhs, T follicular helper cells; IRF5, IFN regulatory factor 5; ABCs, age-associated B cells.

Spontaneous formation of autoimmune germinal center (GC) B cells and the presence of T follicular helper cells (Tfhs) have been shown in several mouse lupus models. These same populations are also present in human SLE (39), and it is thought that the interplay between GC B cells and Tfhs is important for the subsequent generation of pathogenic autoantibodies (40). We found that in mice as young as 8 weeks of age, there was an appreciable population of CD19^+^CD95^+^CD38^–^ cells (GC B cells) in the spleens of *FcγRIIB*^−/−^*Yaa* and *IRF5*^F/+^ mice (Figure 6, A and B). Monoallelic deletion of *IRF5* in B cells dramatically reduced the number of GC B cells, comparable to the reduction observed in mice that have global homozygous and heterozygous *IRF5* deficiency (Figure 6, A and B). Concordantly, we observed a similar marked reduction in splenic Tfhs (CD3^+^CD4^+^CXCR5^+^PD-1^+^) in the *IRF5*^Δ^B mice compared with the *FcγRIIB*^−/−^*Yaa* mice and the littermate *FcγRIIB*^−/−^*Yaa* mice with the *IRF5*-floxed allele alone (Figure 6, C and D).

Age-associated B cells (ABCs) are a population of GC-derived memory B cells that are CD11c^+^T-bet^+^ and are thought to be pathogenic in autoimmune disease (41). It was recently shown that IRF5 expression in B cells is required for the in vitro differentiation of naive B cells to ABCs and that global heterozygous *IRF5* deficiency results in a reduction in ABCs in the SWEF-deficient mouse model in vivo (29). We identified ABCs as CD19^+^B220^+^CD93^–^CD43^–^CD21^–^CD23^–^CD11c^+^T-bet^+^ (Figure 6E; refs. 41, 42) and found a large reduction in the number of splenic ABCs in the *IRF5*^Δ^B mice (Figure 6F). The reduction in the number of ABCs in the *IRF5*^Δ^B mice was similar to the reduction observed in mice with 1 or 2 copies of *IRF5* globally deleted (Figure 6F). Taken together, these data indicate that there was a critical threshold level of *IRF5*, specifically in B cells, that was necessary for the formation of spontaneous GC B cells, Tfhs, and ABCs in the *FcγRIIB*^−/−^*Yaa* lupus model early in disease pathogenesis.

### Monoallelic deletion of IRF5 reduced IL-6 and TNF-α production from B cells and reduced serum IL-6 and TNF-α levels in vivo.

**Figure 7:**
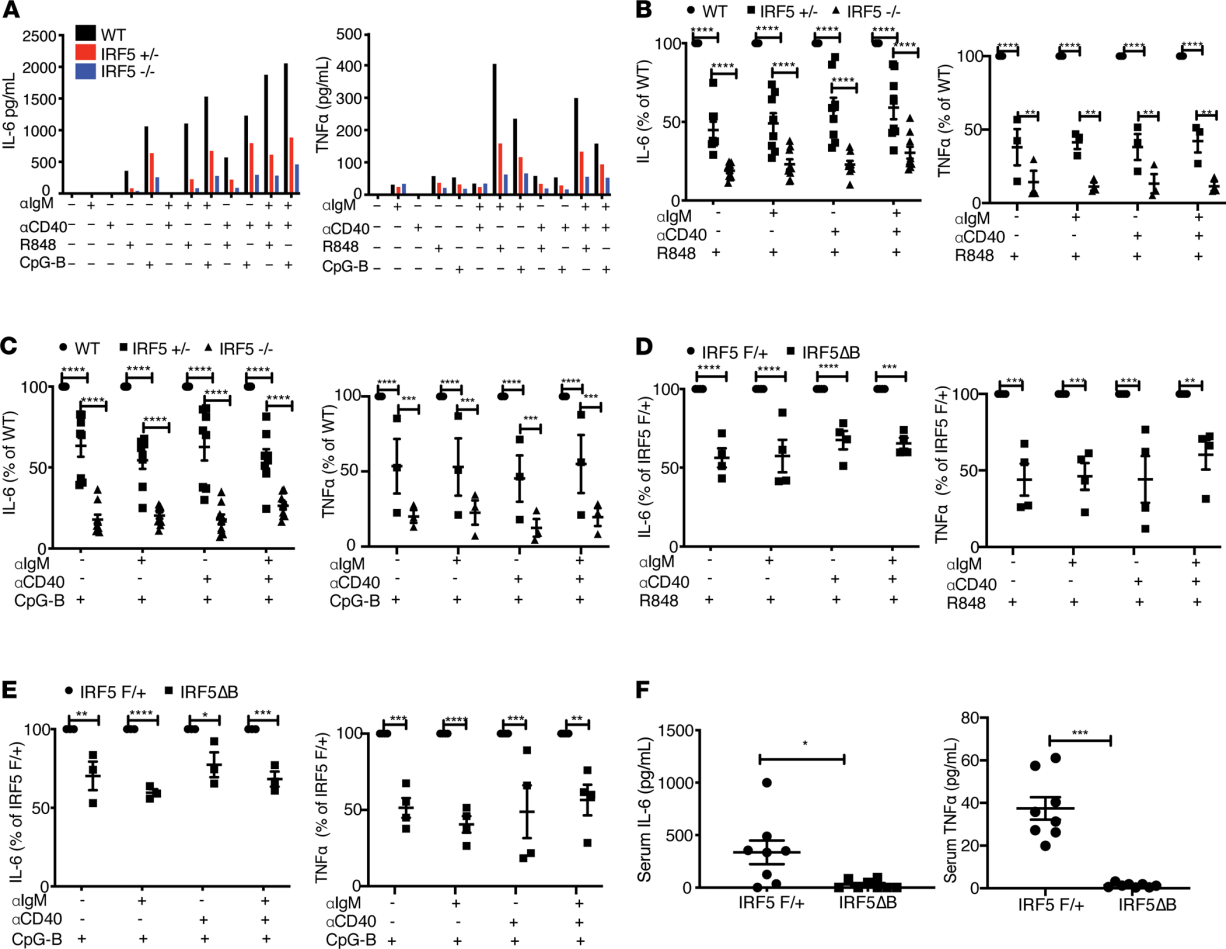
Reduced IRF5 expression in B cells decreases IL-6 and TNF-α production in vitro, and serum IL-6 and TNF-α is reduced in *IRF5^Δ^*B mice. (**A**–**C**) B cells were isolated from the spleens of *FcγRIIB*^−/−^*Yaa* mice at 8–10 weeks of age and stimulated for 24 hours with anti-IgM, anti-CD40, R848, and CpG-B alone or in combination. (**A**) Representative experiments showing mean IL-6 and TNF-α production by B cells from WT, *IRF5*^+/–^, and *IRF5*^–/–^ mice (*n* = 2 for each genotype). (**B** and **C**) IL-6 (*n* = 8) and TNF-α (*n* = 3) production after R848 stimulation (**B**) and CpG-B stimulation (**C**) by B cells from WT, *IRF5*^+/–^, and *IRF5*^–/–^ mice normalized to the WT control in each experiment. (**D** and **E**) IL-6 and TNF-α production by B cells from *IRF5*^Δ^B mice normalized to the littermate *IRF5*^F/+^ control in each experiment (*n* = 4 for each genotype). Data are shown as mean ± SEM and were analyzed using 2-way ANOVA with Tukey’s post hoc test; **P < 0.01, ***P < 0.001, ****P < 0.0001. (**F**) Mean serum IL-6 and TNF-α levels from 5-month-old *IRF5*^Δ^B (*n* = 8) and littermate *IRF5*^F/+^ (*n* = 8) mice. Data are shown as mean ± SEM and were analyzed using 2-tailed, unpaired Welch’s *t* test; *P < 0.05, ***P < 0.001. IRF5, IFN regulatory factor 5.

We next wanted to investigate how IRF5 expression in B cells drives the development of spontaneous GC B cells. B cell–derived IL-6 has been shown to be necessary for the development of spontaneous GCs in a mouse model of lupus and has also been suggested to be important in the development of experimental autoimmune encephalomyelitis (43, 44). B cell–derived soluble TNF is required for the development of GCs in response to T-dependent antigens and for an efficient humoral immune response (45, 46). B cell–derived soluble TNF has also been shown to mediate disease severity in a mouse model of collagen-induced arthritis via control of pathogenic autoantibody production (47). To evaluate how *IRF5* monoallelic deficiency might impact B cell–intrinsic IL-6 and TNF-α production induced by the different pathways critical for GC B cell formation, we isolated B cells from 8-week-old *FcγRIIB*^−/−^*Yaa*, *FcγRIIB*^−/−^ *Yaa* *IRF5*^+/−^, or *FcγRIIB*^−/−^ *Yaa* *IRF5*^−/−^ mice and stimulated the B cells with various combinations of anti-IgM antibody to model B cell receptor (BCR) activation, anti-CD40 antibody to model T cell costimulation, and the TLR7 ligand R848 or TLR9 ligand CpG-B. We found that a modest degree of IL-6 and TNF-α was induced by R848 or CpG-B alone. A substantial increase in IL-6 and TNF-α production was observed with R848 or CpG-B in combination with anti-IgM (Figure 7A). There was a gene dosage–dependent impact of *IRF5* on IL-6 and TNF-α in that heterozygous deficiency of *IRF5* reduced IL-6 and TNF-α production by 40%–60% in all conditions tested and homozygous deletion reduced IL-6 and TNF-α by 70%–80% (Figure 7, B and C). Notably, B cells from mice with B cell–specific heterozygous deletion of *IRF5* by Mb1cre showed a similar reduction in IL-6 and TNF-α as observed in B cells from mice with germ line heterozygous deficiency of *IRF5* (Figure 7, D and E). IL-12p40 production was also reduced in *IRF5* heterozygous B cells in response to most stimuli combinations, but effects on RANTES, MIP-1α, and MIP-1β were more variable (Supplemental Figure 5).

We next evaluated if B cell–specific deletion of IRF5 impacted the level of serum IL-6 and/or TNF-α in the *FcγRIIB*^−/−^*Yaa* model. B cell–specific deletion of IL-6 has been shown to reduce serum IL-6 in another lupus model (43). We found that serum levels of both IL-6 and TNF-α were indeed decreased in 5-month-old *IRF5*^Δ^B mice compared with the *FcγRIIB*^−/−^*Yaa* controls (Figure 7F). These data suggest that there was a synergistic effect of both TLR7 and TLR9 signaling with BCR signaling on the production of IL-6 and TNF-α and that B cell–intrinsic IRF5 played a critical role in mediating the induction of IL-6 and TNF-α in all combinations of stimuli.

### TLR7 and BCR stimulation synergized in the induction of IRF5 phosphorylation.

**Figure 8:**
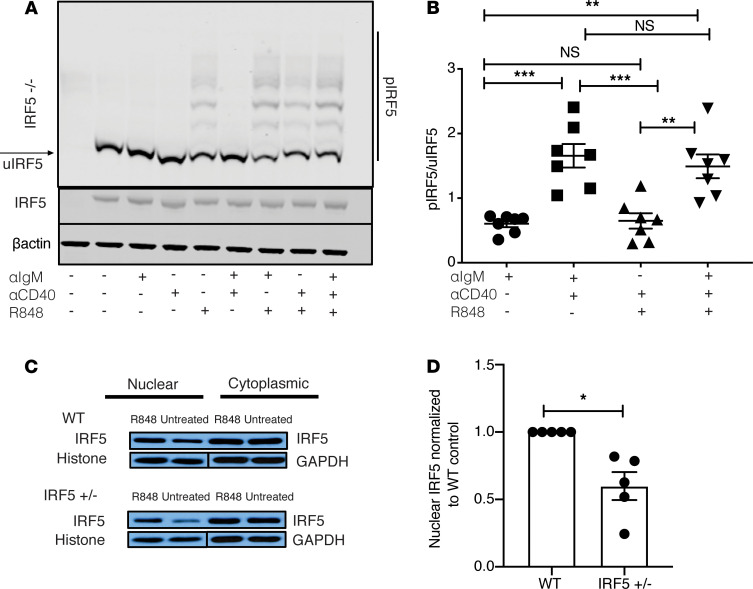
TLR7 signaling is required for IRF5 phosphorylation, and IRF5 nuclear translocation is reduced in B cells from *FcγRIIB^−/−^Yaa IRF5^+/–^* mice. (**A** and **B**) B cells were isolated from the spleens of *FcγRIIB*^−/−^*Yaa* mice at 8–10 weeks of age. (**A**) B cells were stimulated with anti-IgM, anti-CD40, and R848 alone or in combination for 2 hours and the protein lysate analyzed using phospho-Tag gel (upper panel) or standard gel (lower panels). B cells isolated from an *IRF5*-deficient (*IRF5*^–/–^) mouse are shown in the first lane. p-IRF5 denotes phosphorylated IRF5. A representative example of 7 individual experiments is shown. (**B**) Ratio of p-IRF5 to unphosphorylated IRF5 (u-IRF5). Intensity of p-IRF5 was normalized to the intensity of unphosphorylated IRF5 (lowest band of IRF5 on p-Tag gel as shown in **A**) (*n* = 7). (**C** and **D**) B cells from *FcγRIIB*^−/−^*Yaa* (WT) and *FcγRIIB*^−/−^*Yaa* *IRF5*^+/–^ mice were stimulated for 2 hours with R848, or not stimulated (untreated), and IRF5 was probed in the nuclear and cytoplasmic fractions. (**C**) A representative experiment of 4 individual experiments is shown. (**D**) Ratio of IRF5 expression in nucleus relative to the WT after R848 stimulation; nuclear IRF5 intensity in each sample was first normalized to its own loading control (histone; *n* = 5). Data are shown as mean ± SEM and were analyzed using 1-way ANOVA with Tukey’s post hoc test; *P < 0.05, **P < 0.01, ***P < 0.001. IRF5, IFN regulatory factor 5.

Phosphorylation of IRF5 is necessary for IRF5 dimerization and nuclear translocation (48). To determine whether the increased IL-6 and TNF-α production observed with TLR7 and BCR costimulation might be mirrored by changes in IRF5 phosphorylation, we stimulated isolated B cells from *FcγRIIB*^−/−^*Yaa* mice with the different combinations of anti-IgM, R848, and anti-CD40. We chose to use R848 because TLR7 gene dosage is essential for the accelerated disease in the *FcγRIIB*^−/−^*Yaa* mice and TLR7 is important for pathogenesis in other mouse models of lupus (16, 49). We did not detect any phosphorylation of IRF5 after stimulation with anti-IgM alone or anti-CD40 alone, consistent with the lack of IL-6 and TNF-α production observed in Figure 7A. However, we readily detected phosphorylation after stimulation with R848 alone for 2 hours (Figure 8A). The ratio of phosphorylated to unphosphorylated IRF5 was significantly increased 3-fold with the addition of anti-IgM to R848 (Figure 8B). Interestingly, the addition of anti-CD40 stimulation did not increase IRF5 phosphorylation in any of the stimulus combinations. Moreover, we demonstrated that B cells from C57BL/6 mice harboring the kinase dead mutation of IL-1 receptor–associated kinase 4 (IRAK4) completely lacked IRF5 phosphorylation in all stimulation conditions (Supplemental Figure 6A). These data indicate that TLR7 stimulation alone was sufficient to induce IRF5 phosphorylation in B cells and that the BCR and TLR7 synergized to increase IRF5 phosphorylation.

### TLR7-induced IRF5 phosphorylation and nuclear translocation were reduced in B cells with monoallelic deletion of IRF5.

Because TLR7-induced IL-6 and TNF-α production were reduced in B cells with monoallelic deletion of *IRF5* (Figure 7, A, B, and D), we hypothesized that this would be associated with a reduction in IRF5 nuclear translocation. We first examined the extent of IRF5 phosphorylation in splenic B cells from *FcγRIIB*^−/−^*Yaa* WT and *IRF5*^+/−^ mice 2 hours after stimulation with anti-IgM, anti-CD40, and R848 alone or in combination. We found that the general pattern of IRF5 phosphorylation, as assessed by phospho-Tag gel, and the ratio of phosphorylated to unphosphorylated IRF5 did not differ between the groups (Supplemental Figure 6, B and C). However, because the total amount of IRF5 in B cells from the *FcγRIIB*^−/−^ *Yaa* *IRF5*^+/−^ mice was about half of that in the WT mice, we infer that the total amount of phosphorylated IRF5 was less in the *IRF5*^+/−^ mice under these stimulation conditions. More importantly, we directly compared the ratio of nuclear IRF5 expression in B cells from the WT and *IRF5*^+/−^ mice after R848 stimulation and found that nuclear IRF5 expression was reduced by approximately half in the *IRF5*^+/−^ mice (Figure 8, C and D).

### IRF5 expression was increased in activated B cells in vitro and in GC B cells, splenic plasmablasts, and bone marrow plasma cells in vivo.

**Figure 9:**
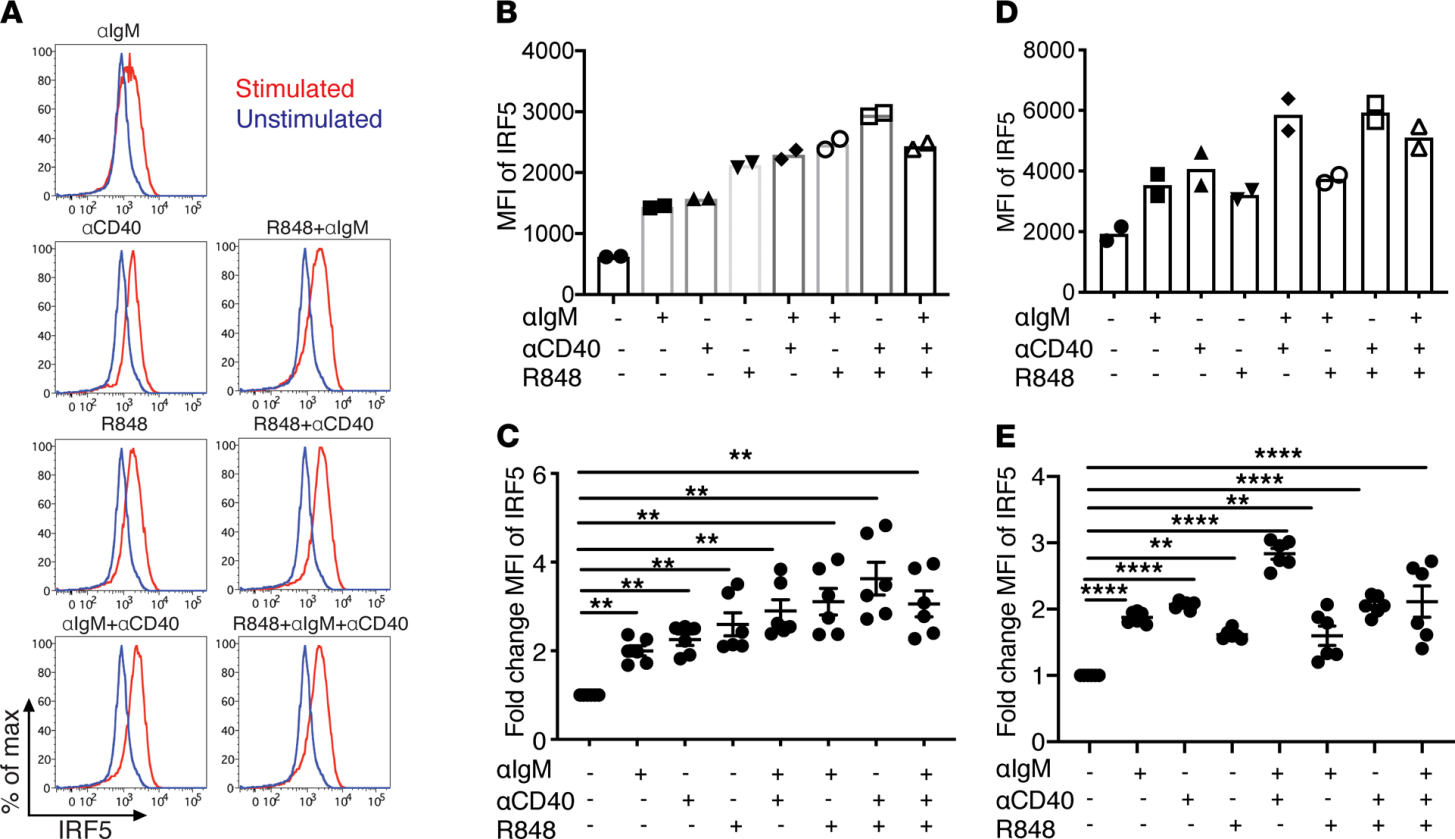
IRF5 expression is increased in activated B cells in vitro. (**A**–**E**) Splenic B cells were isolated from 8- to 10-week-old *FcγRIIB*^−/−^*Yaa* or C57BL/6 mice and were either not stimulated or stimulated with anti-IgM, anti-CD40, and R848 alone or in combination for 24 hours. (**A**) Intracellular IRF5 levels were measured using flow cytometry. A representative experiment of 6 individual experiments using B cells from *FcγRIIB*^−/−^*Yaa* mice is shown. (**B** and **D**) MFI of IRF5 with and without stimulation in B cells from *FcγRIIB*^−/−^*Yaa* mice (**B**) and C57BL/6 mice (**D**) (*n* = 2 per strain). (**C** and **E**) Fold change of IRF5 expression normalized to unstimulated control in B cells from *FcγRIIB*^−/−^*Yaa* mice (**C**) and C57BL/6 mice (**E**) (*n* = 6 per strain). Data are shown as mean ± SEM and were analyzed using 1-way ANOVA with Tukey’s post hoc test; **P < 0.01, ****P < 0.0001. IRF5, IFN regulatory factor 5.

Our data in Figure 3 and Figure 6 suggest that there was a critical threshold level of IRF5 required for the generation of autoimmune GC B cells and plasma cells. We therefore hypothesized that IRF5 expression levels might be dynamically regulated after B cell activation. To determine if the expression level of IRF5 changes after B cell activation, we stimulated naive B cells isolated from *FcγRIIB*^−/−^*Yaa* mice using various combinations of anti-IgM, anti-CD40, and R848 and measured IRF5 expression levels by intracellular flow cytometry after 24 hours. In contrast to their lack of effect on IRF5 phosphorylation, anti-IgM and anti-CD40 alone increased IRF5 expression about 2-fold, similar to the increase induced by R848 alone (Figure 9, A–C). The various stimulus combinations induced a greater increase in IRF5 expression than the individual stimuli alone. To determine whether these findings were specific to B cells from *FcγRIIB*^−/−^*Yaa* mice, we repeated these studies using naive B cells isolated from C57BL/6 mice and found a similar increase in IRF5 expression in C57BL/6 B cells (Figure 9, D and E). This indicates that the increase in IRF5 expression observed in B cells under these stimulation conditions was not a unique feature of *FcγRIIB*^−/−^*Yaa* B cells only.

**Figure 10:**
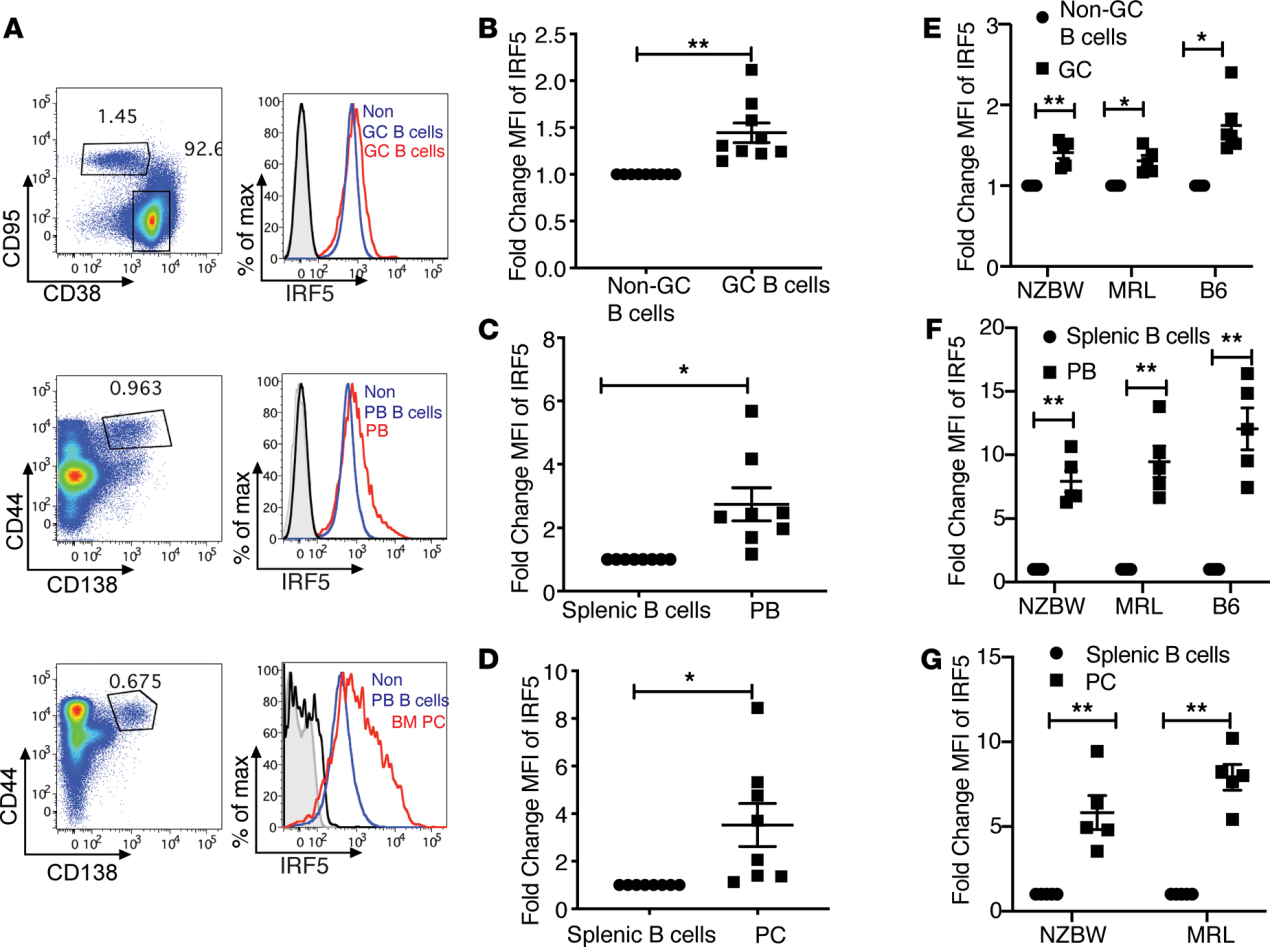
IRF5 expression is increased in GC B cells, splenic PBs, and BM PCs in vivo. (**A**–**G**) Flow cytometry performed on splenocytes and bone marrow from *FcγRIIB*^−/−^*Yaa* mice at 8–10 weeks of age. (**A**) Representative examples of IRF5 expression in GC B cells (CD38^–^CD95^+^CD19^+^) and non-GC B cells (CD38^+^CD95^–^; upper panel); PBs (CD44^+^CD138^+^) and CD19^+^CD138^–^ B cells (non-PBs; middle panel); and BM PCs (CD44^+^ CD138^+^) and non-PB B cells from spleens (lower panel). (**B**) Fold change in IRF5 expression in GC B cells normalized to non-GC B cells (*n* = 9). (**C**) Fold change in IRF5 expression in PBs normalized to non-PBs (*n* = 8). (**D**) Fold change in IRF5 expression in PCs normalized to splenic non-PBs (*n* = 8). (**E**–**G**) IRF5 expression in NZB/W mice (*n* = 6), MRL/*lpr* mice (*n* = 6), and C57BL/6 (*n* = 6) mice immunized with 4-hydroxy-3-nitrophenylacetyl coupled to chicken γ-globulin. (**E**) Fold change of IRF5 expression in GC B cells. (**F**) Fold change of IRF5 expression in PBs. (**G**) Fold change of IRF5 expression in plasma cells. Black histogram shows isotype control in non-GC B cells, non-PBs, or BM PCs; gray-tinted histogram shows isotype control in GC B cells, PBs, or BM PCs; blue histogram shows IRF5 expression in non-GC B cells and non-PBs; red histogram shows IRF5 expression in GC B cells, PBs, or BM PCs. Data are shown as mean ± SEM and were analyzed using 2-tailed, unpaired Welch’s *t* test; *P < 0.05, **P < 0.01. IRF5, IFN regulatory factor 5; GC, germinal center; PBs, plasmablasts; BM PCs, bone marrow plasma cells.

The increase in IRF5 expression after activation with anti-IgM, anti-CD40, and R848 in vitro suggested the possibility that IRF5 might be increased in GC B cells in *FcγRIIB*^−/−^*Yaa* mice in vivo, because TLR7 activation is thought to be important for spontaneous GC formation in other lupus models (50). To test this hypothesis, we evaluated IRF5 expression in splenic GCs of 8- to 10-week-old *FcγRIIB*^−/−^*Yaa* mice. We observed an approximately 1.5-fold increase in the MFI of IRF5 in GC B cells compared with non-GC B cells (Figure 10, A and B). Next, we evaluated the levels of IRF5 in plasmablasts because these cells are thought to be derived from either the GC or an extrafollicular response (1) and because it was recently reported that human plasmablasts express higher levels of IRF5 compared with naive B cells (35). We observed a roughly 2.5-fold increase in the MFI of IRF5 in plasmablasts compared with non–plasmablast B cells (CD19^+^; Figure 10, A and C). Furthermore, we did a similar analysis comparing the splenic CD19^+^ B cells with plasma cells in the bone marrow and observed an increase in IRF5 MFI in plasma cells at least as large as that observed in the splenic plasmablasts (Figure 10, A and D).

To determine whether our findings were specific to the *FcγRIIB*^−/−^*Yaa* model or were more generally relevant, we measured changes in IRF5 expression in GC B cells, plasmablasts, and plasma cells in 2 additional mouse lupus models, (NZBxNZW)F1 and MRL-*lpr*. We found that IRF5 expression was increased in these models at least as much as had been observed in the *FcγRIIB*^−/−^*Yaa* model (Figure 10, E–G). In addition, to determine whether our findings were restricted to lupus models or whether they might reflect GC responses more broadly, we measured changes in IRF5 expression in GC B cells and plasmablasts in C57BL/6 mice 14 days after immunization with the hapten 4-hydroxy-3-nitrophenylacetyl coupled to chicken γ-globulin. We found an increase in IRF5 expression in these cell types similar to that observed in the 3 lupus models (Figure 10, E and F).

Taken together, these data suggest that IRF5 expression levels were dynamically regulated in the GC compartment as well as during plasmablast differentiation and that this increased IRF5 expression was maintained in long-lived plasma cells.

## Discussion

Global homozygous or heterozygous deficiency of *IRF5* markedly reduces disease severity in mouse lupus models (5–9). However, it is not known which IRF5-expressing cell type(s) is involved in lupus pathogenesis. In this report, we demonstrate that heterozygous deficiency of *IRF5* in B cells resulted in a marked reduction in lupus disease manifestations. In contrast, similar reduction of IRF5 expression in macrophages, monocytes, and neutrophils did not reduce disease severity. Although these findings do not exclude a role for IRF5 in other cell types in disease pathogenesis, they do demonstrate that there is a critical threshold level of IRF5 expression in B cells that was absolutely required.

We previously reported that heterozygous deficiency of *IRF5* reduces disease in the *FcγRIIB*^−/−^*Yaa* mouse lupus model (5). Subsequently, it was demonstrated that global heterozygous deficiency of *IRF5* also reduces disease in the Lyn-deficient mouse lupus model, the *gld*.apoE-deficient mouse lupus model, and in the Swap70-deficient Def6-deficient mouse lupus model (10, 28, 29). In the Swap70-deficient Def6-deficient model, the deletion of the second *IRF5* allele specifically in CD21-expressing cells or in CD11c-expressing cells does not lead to further reductions in disease manifestations. In this report, we identified B cells as a critical cell type in which heterozygous deficiency of *IRF5* was protective, and thus we sought to uncover possible mechanisms that might explain the profound protective effect of a 50% reduction in IRF5 expression in B cells. We found that IL-6 and TNF-α production by B cells from *IRF5*-heterozygous mice was reduced by about half in response to combinations of stimuli that are thought to be involved in B cell activation in the GC in lupus (51). In contrast, the expression of costimulatory molecules involved in B cell–T cell interactions and GC B cell activation, such as CD80, CD86, MHC class II, and ICOS-ligand, did not differ between B cells from *IRF5*-WT and *IRF5*-heterozygous mice (Supplemental Figure 7). B cell–intrinsic IL-6 and TNF-α production have been shown to be important in GC formation in mice immunized with T-dependent antigens and in GC formation and disease pathogenesis in lupus and other autoimmune models (43–47). It is thus plausible that the reduction in IL-6 and TNF-α production observed in our studies may explain, at least in part, the reduction in disease manifestations seen in the B cell–specific *IRF5*-heterozygous mice.

In the inactivated state, IRF5 resides in the cytoplasm as a monomer. IRF5 activation requires phosphorylation of the monomer, leading to either IRF5 homodimerization or heterodimerization of IRF5 with other IRF or NF-κB family members, followed by nuclear translocation and the induction of target gene transcription (17). We found that the amount of IRF5 in the nucleus after TLR7 activation was approximately 50% lower in B cells from *IRF5*-heterozygous *FcγRIIB*^−/−^*Yaa* mice than in B cells from *IRF5*-sufficient *FcγRIIB*^−/−^*Yaa* mice. Mechanistically, this may explain the reduced production of IL-6 and TNF-α by the *IRF5*-heterozygous B cells.

The *IRF5* polymorphisms associated with an increased risk of developing SLE and other autoimmune diseases are in the *non*coding region of the *IRF5* gene and show some association with increased IRF5 expression and/or functional change (3, 20). We found that BCR activation alone or CD40 activation alone increased IRF5 protein about 2-fold but did not induce IRF5 phosphorylation. Thus, increased IRF5 expression, per se, did not result in IRF5 activation. In contrast, TLR7 activation alone induced both increased IRF5 expression and IRF5 phosphorylation, and BCR activation, although not able to induce IRF5 phosphorylation independently, synergized with TLR7 activation to further increase both IRF5 expression and phosphorylation. These findings were not just restricted to B cells from an autoimmune disease–prone background but were also observed in B cells from C57BL/6 mice. Intriguingly, we also observed that IRF5 phosphorylation in all stimulation conditions was IRAK4 dependent. Consistent with these in vitro findings, we observed an increase in IRF5 expression in GC B cells in vivo early in disease development, prior to the appearance of overt signs of autoimmunity, with even larger increases evident in plasmablasts and bone marrow plasma cells. We corroborated these findings in 3 different lupus mouse models and in C57BL/6 mice immunized with a T-dependent antigen, suggesting that the increase in IRF5 in these compartments during B cell activation could have been a general feature in B cell biology.

IRF5 exerts a number of effects in B cells, including class-switching to IgG2a/IgG2c by binding to the IgG2a promoter, regulation of the transcription factor Blimp1, and regulation of T-bet–expressing ABCs (6, 23, 29). Although these B cell–intrinsic effects likely make significant contributions to disease pathogenesis, our data suggest that there were also critical roles for B cell–intrinsic IRF5 more proximally in the autoimmune response. The generation of high-affinity autoantibodies and long-lived plasma cells in lupus is dependent on the development of a robust GC response, with TLR7 and TLR9 signaling in B cells likely being required for this response (51). We found a dramatic reduction in the number of GC B cells prior to the onset of overt autoimmune disease in the mice with heterozygous deficiency of *IRF5* in B cells. This was accompanied by an equally marked reduction in the number of Tfhs, a cell type that provides the signals to B cells that are required for GC formation, affinity maturation, and the development of most high-affinity autoantibodies and memory B cells (52, 53). This suggests that B cell–intrinsic IRF5 may be required for the generation of Tfhs in our lupus model. Early Tfh differentiation is regulated by IL-6, ICOS, IL-2, and T cell receptor signal strength in mouse models (52). This early Tfh cell differentiation is generally thought to be the result of naive T cell interactions with DCs. However, B cells can be the dominant antigen-presenting cells that activate naive CD4^+^ T cells in certain circumstances, and B cells activated by TLR9 and TLR7 ligands in the context of viral infection are sufficient to induce Tfh development in the absence of DCs (54).

In summary, we have demonstrated that there was a critical threshold level of IRF5 in B cells that was required for disease development in the *FcγRIIB*^−/−^*Yaa* mouse lupus model and that IRF5 nuclear translocation was substantially reduced in B cells from *IRF5*-heterozygous mice after TLR7 activation. We also showed in multiple lupus models and in immunization studies that the level of IRF5 expression was dynamically regulated throughout the B cell activation process, increasingly and progressively from GC B cells to mature plasma cells. Furthermore, IRF5 phosphorylation was increased in a synergistic manner by activation signals believed to be important in the generation of autoreactive GC B cells. The fact that a 50% reduction in B cell IRF5 expression was sufficient to largely abrogate disease development suggests that targeting IRF5 in B cells may have been an effective therapeutic approach in lupus.

## Methods

### Generation of IRF5-floxed mice.

The vector to target *IRF5* was pEZ-Frt-LoxP-DT (courtesy of Klaus Rajewsky, Max Delbrück Center for Molecular Medicine in the Helmholtz Association, Berlin, Germany). Three DNA fragments of the C57BL/6 mice *IRF5* locus were inserted into the cloning site of the vector pEZ-Frt-LoxP-DT: 1.6 kb of the region upstream of the *IRF5* exon 3, 2 kb of the region between exon 3 to exon 6, and 2.8 kb of the region downstream of exon 6 (Supplemental Figure 2). The linearized vector was electroporated into embryonic stem (ES) cells derived from C57BL/6 mice in the Transgenic Mouse Core at Brigham and Women’s Hospital (Boston, Massachusetts, USA), and correctly targeted ES cell colonies were identified by PCR. Selected ES cells were injected into blastocysts. Chimeras were bred with C57BL/6 mice to obtain mice with the conditional *IRF5* allele in the germ line as determined by PCRs. The mice were further crossed with ROSA26:FLPe knockin mice (Jackson Laboratory, 9086) to delete the neomycin-resistant gene between Frt sites. *IRF5* *LoxP* positioning was confirmed by PCR and Southern blotting analysis.

### Animals.

WT (*IRF5*^+/+^) *FcγRIIB*^−/−^*Yaa*, *IRF5*^–/–^ *FcγRIIB*^–/–^*Yaa*, and *IRF5*^+/–^ *FcγRIIB*^–/–^*Yaa* mice were all generated as previously described (5). *FcγRIIB*^–/–^*Yaa* *Mb1*^cre/+^ *IRF5*^fl/+^ (*IRF5*^Δ^B) were generated by crossing *IRF5*^fl/fl^ mice on a *FcγRIIB*^–/–^*Yaa* background. Mice that expressed cre under the direction of the Ig-α promoter (mb1-cre mice) were provided by Michael Reth (University of Freiburg, Freiburg, Germany; ref. 32) and were also crossed on a *FcγRIIB*^–/–^*Yaa* background. *Mb1*^cre/+^ *FcγRIIB*^–/–^*Yaa* males were then crossed to *FcγRIIB*^–/–^ *IRF5*^fl/fl^ females. The final experimental and littermate control mice were generated by breeding *Mb1*^cre/+^ *FcγRIIB*^−/−^*Yaa* males with *FcγRIIB*^−/−^ *IRF5*^fl/+^ females. B6.SJL-Ptprc^a^ Pepc^b^/BoyJ (CD45.1) mice were purchased from Jackson Laboratory. Mice that expressed cre under the direction of the LysM promoter (LysMcre mice) were purchased from Jackson Laboratory (B6.129P2-*Lyz2^tm1(cre)Ifo^*/J; strain number 004781; ref. 37). The final experimental and littermate control mice were generated in a similar manner to the Mb1cre study detailed above, except that the *LysM*^cre/+^ *FcγRIIB*^–/–^*Yaa* males were bred with *FcγRIIB*^–/–^ *IRF5*^fl/fl^ females, not *FcγRIIB*^–/–^ *IRF5*^fl/+^ females. Mice deficient in *IRAK-4*^KN/KN^ were provided by Shizuo Akira (Osaka University, Osaka, Japan; ref. 55). (NZBxNZW)F1 mice and MRL-*lpr* mice were purchased from Jackson Laboratory (stock numbers 100008 and 000485, respectively). All mice used in this study, apart from (NZBxNZW)F1 and MRL-*lpr*, were backcrossed more than 9 generations on a C57BL/6 background.

### Bone marrow chimera generation.

Bone marrow cells were isolated from *FcγRIIB*^–/–^*Yaa* *IRF5*^+/+^ and *FcγRIIB*^–/–^*Yaa* *IRF5*^–/–^ donors and washed once in PBS followed by RBC lysis using RBC lysing buffer (MilliporeSigma, R7757). Donor marrow cells (10^6^ cells per mouse) were injected into the tail veins of B6.SJL-Ptprc^a^ Pepc^b^/BoyJ (CD45.1) recipient mice after cesium irradiation of recipient mice with 2 doses of 500 rad separated by 3 to 4 hours. Recipient mice were subsequently placed in irradiated cages, and water was supplemented with sulfamethoxazole/trimethoprim (Hi-Tech Pharmaceuticals) (at 2/0.4 mg/mL). Immune cell engraftment was subsequently confirmed using flow cytometry of PBMCs and antibodies specific for CD45.1 (clone A20; BioLegend) and CD45.2 (clone 104; BioLegend).

### Immunization.

C56BL/6 mice (8–10 weeks old) were immunized i.p. with 50 g 4-hydroxy-3-nitrophenylacetyl coupled to chicken γ-globulin at a substitution rate of 16 (Biosearch Technologies) in alum (56). Spleens were harvested on day 14.

### B cell isolation and stimulation.

Resting B cells were purified from the spleens of WT *FcγRIIB*^–/–^*Yaa*, *IRF5*^–/–^ *FcγRIIB*^–/–^*Yaa*, *IRF5*^+/–^ *FcγRIIB*^–/–^*Yaa*, *IRF5*^F/+^ *FcγRIIB*^–/–^*Yaa*, and *IRF5*^Δ^B mice by negative selection using MACS cell separation with anti-CD43 beads (Miltenyi Biotec). Cells were plated at 1–2 *×* 10^5^ per well in duplicate or triplicate in RPMI 1640 supplemented with 10% FBS, 2 mM l-glutamine, 50 μM 2-mercaptoethanol, 100 U/mL penicillin, and 100 μg/mL streptomycin at 37°C and stimulated with 0.05 μM R848 (InvivoGen), 10 μg/mL anti-IgM [F(ab′)_2_ fragment; μ chain specific; Jackson ImmunoResearch], and 2 μg/mL anti-CD40 antibody (clone HM40-3, BD Biosciences) alone or in combination. Supernatants were collected 24 hours later and cytokines evaluated by ELISAs (R&D Systems and BioLegend) and ProcartaPlex multiplex (Invitrogen). Cells were evaluated by flow cytometry for activation markers and intracellular levels of IRF5.

### Western blot and phospho-tag immunoblot analysis.

Splenic B cells were lysed in RIPA buffer (MilliporeSigma) that included phosphatase and protease inhibitors (MilliporeSigma). For standard Western blot analysis, samples were separated on a 4%–12% Bis-Tris gel (Invitrogen) using MOPS buffer (Invitrogen). For phospho-tag immunoblot analysis, SDS-PAGE was performed using SuperSep Phos-tag 7.5% precast gel (Fujifilm Wako Chemicals USA Corporation). Samples were transferred to nitrocellulose membranes using iBlot (Invitrogen) and probed with anti-IRF5 antibody (catalog ab181553, Abcam) and anti–β-actin antibody (clone 8H10D10, Cell Signaling Technology). Samples were imaged using the Odyssey CLx (LI-COR) and quantified using ImageStudio.

### Preparation of cytosolic and nuclear protein fractions.

Cytosolic and nuclear protein fractions were prepared using a slight modification of the protocol described by Liou et al. (57). Splenic B cells were washed once with cold PBS and resuspended in buffer A (10 mM HEPES [pH 7.9], 1.5 mM MgCl_2_, 10 mM KCl, and 0.5 mM DTT) with 0.1% Triton X-100, phosphatase inhibitors (MilliporeSigma), and protease inhibitors (Calbiochem). Cells were lysed on ice for 10 minutes. After centrifugation at 6000*g* for 15 minutes at 4ºC, the supernatant was saved as the cytosolic fraction. The nuclear pellet was washed twice with buffer A to remove any residual cytosolic fraction proteins and then resuspended in buffer C (20 mM HEPES [pH 7.9], 25% glycerol, 0.42 M NaCl, 1.5 mM MgCl_2_, 0.2 mM EDTA, and 0.5 mM DTT) with protease inhibitors on ice. The nuclear pellet was sonicated briefly before incubation on ice for 20 minutes. The insoluble debris were removed by centrifugation at 15,000*g* for 15 minutes at 4ºC, and the supernatant was saved as the nuclear fraction. The protein concentration was determined with Pierce BCA Protein Assay Kit (Life Technologies).

### Flow cytometry.

Splenocytes were labeled with antibodies against CD4 (clone GK1.5, BD Biosciences), CD8 (clone 53-6.7, BioLegend), and CD3 (clone 17A2, BD Biosciences) to identify T cell populations; against CD19 (clone 1D3, BD Biosciences) and B220 (clone RA3-6B2, BD Biosciences) to identify B cells; and against CD44 (clone IM7, BD Biosciences) and CD62L (clone MEL-14, BioLegend) to identify naive T cells, activated T cells, and memory T cells. Biotin-labeled anti-CXCR5 (clone 2G8, BD Biosciences) was used in combination with streptavidin-BV711 (BD Biosciences, catalog 563262), CD3, PD-1 (clone J43, BD Biosciences), and CD4 to identify Tfhs. ABC B cells were identified by first gating on CD19 and B220, then gating out CD43^+^ (clone S7, BD Biosciences) and CD93^+^ (clone AA4.1, BD Biosciences) cells, followed by gating on CD21^–^ (clone 7G6, BD Biosciences) and CD23^–^ (clone B3B4, BD Biosciences) negative cells. Finally, CD11c (clone HL3, BD Biosciences) and Tbet (clone eBio4B10, eBioscience) delineated Tbet^+^ ABCs. The FoxP3 fix/perm from eBioscience (catalog 00-5523-00) was used for the intracellular staining of Tbet. For staining of spleen plasmablasts, antibodies against CD3, CD19, B220, CD44, and CD138 (clone 281-2, BD Biosciences) were used. For staining of bone marrow plasma cells, antibodies against CD4, CD8, Gr-1 (clone RB6-8C5, BioLegend), and F4/80 (clone BM8, BioLegend) were used to gate out non-B cells, and plasma cells [CD138 (281-2)^+^B220^−^] were identified using antibodies against B220 and CD138. Bone marrow plasma cells were also identified using same stain as used for spleens. Antibodies against CD19, CD38 (clone 90, BioLegend), CD95 (clone Jo2, BD Biosciences), and B220 were used to define germinal center B cells. Antibodies against CD80 (clone 16-10A1, BioLegend), CD86 (clone GL-1, BioLegend), CD275 (clone HK5.3, BioLegend), and MHCII (clone M5/114.15.2, eBioscience) were used to evaluate activation. B cells were first gated on CD19^+^ and live cells (live/dead, catalog 65-0866-14, eBioscience). Immunofluorescence was measured with an LSRII (BD Biosciences). Sorting experiments were done on BD FACSAria III (BD Biosciences). The data were analyzed using FlowJo software (Tree Star).

### Intracellular flow cytometry.

B cells or splenocytes were first stained with extracellular antigens. Cells were then fixed for 15 minutes at room temperature with BioLegend fixative. Cells were then washed with BioLegend permeabilize buffer, and then cells were incubated with anti-IRF5 (clone W16007B, BioLegend) or isotype control (R&D Systems, catalog IC006P) diluted in 1× perm buffer. To identify neutrophils, monocytes, and pDCs, splenocytes were stained with antibodies against CD11b (M1/70), Ly6c (HK1.4), Ly6G (1A8), PDCA1 (eBio927), CD19, and CD3.

### Histology.

At the end of the experiment at 4–5 months of age, kidneys were harvested from *FcγRIIB*^–/–^*Yaa* *IRF5*^F/+^ control and *FcγRIIB*^−/−^*Yaa* *IRF5*^Δ^B mice and fixed in formalin or snap-frozen in OCT compound. Formalin-fixed and paraffin-embedded kidneys were sectioned at 8 μm and then stained with H&E. Kidney disease was assessed by a blinded investigator. To this end, randomly selected areas of cortex were examined, and at least 50 glomeruli from each animal were scored. Glomerular and interstitial disease were evaluated as previously described (5, 58, 59).

### IHC.

Mouse kidneys were snap-frozen in OCT (Tissue-Tek, Sakura Finetek) and stored at –80°C. Seven-micrometer cryosections were cut and fixed with methanol and acetone (1:1), blocked with 1% BSA, and then stained with Alexa Fluor 594–conjugated donkey anti-mouse IgG (Invitrogen, catalog A21203) and FITC-conjugated goat anti-mouse C3 (Cappel Laboratories, catalog 0855500) at 4°C overnight. After washing with Tris-buffered saline, the stained sections were scanned with a Nikon Deconvolution Wide-Field Epifluorescence System. Fluorescence intensity, representing IgG and C3 deposition, was measured using ImageJ (NIH) and analyzed with GraphPad Prism. Representative images were acquired using a Zeiss LSM 710 confocal microscope.

### Serological assays.

IgG isotypes and IgM were measured by ELISA established using antibodies from BD Biosciences and Southern Biotech. ANA titer was measured by immunofluorescence using HEp2-coated slides (INOVA Diagnostics, Inc.) as described (5, 59). The antinucleosome antibody ELISA was developed using nucleosome antigen ATN02-05 (2 μg/mL; Arotec Diagnostics Limited) and HRP-conjugated anti–mouse IgG antibody (1:3000, MilliporeSigma, catalog A8924). Anti-nucleosome antibody (clone PL2-3, gift from Marc Monestier, Temple University, Philadelphia, Pennsylvania, USA) was used as the positive control for quantification. The anti-Sm/RNP ELISA was developed using Sm/RNP antigen ATR01 (2 μg/mL; Arotec Diagnostics Limited) and HRP-conjugated anti–mouse IgG antibody (1:3000, MilliporeSigma). Anti-Sm/RNP antibody (Y2) was used as the positive control for quantification.

### Statistics.

*P* values were calculated using a 2-tailed, unpaired Welch’s *t* test, or a 1-way ANOVA with Tukey’s post hoc test. *P* values less than 0.05 were considered significant. Statistics were calculated using GraphPad Prism.

### Study approval.

All animal studies were performed under protocols that were approved by the IACUC at Boston University and the University of Massachusetts Medical School.

## Author contributions

AP, KY, UK, RGB, and IRR designed the study. AP, KY, ACB, VS, PS, KN, BKH, YX, YT, and RGB performed the experiments and analyzed the data. GAV optimized experimental protocols. AP and IRR wrote the manuscript. AP, KY, VS, PS, BKH, GAV, YX, UK, YT, RGB, and IRR read and commented on various drafts of the manuscript. For the 2 co–first authors, the assignment of authorship order was made after discussion with, and with the full agreement of, both co–first authors. The assignment was based on their overall contributions to the various components of the experimental work and manuscript preparation, recognizing that both co–first authors made very substantial and critical contributions to the manuscript.

## Supplementary Material

Supplemental data

## Version 1 07/01/2021

In-Press Preview

## Version 2 08/09/2021

Electronic publication
