# Supplementary material for: Monoallelic IRF5 deficiency in B cells prevents murine lupus
Source: JCI Insight. 2021 Aug 9;6(15):e141395. doi: 10.1172/jci.insight.141395 (PMC8410043; doi:10.1172/jci.insight.141395)
Supplement: Supplemental data [file jciinsight-6-141395-s187.pdf]

Supplemental Figure 1

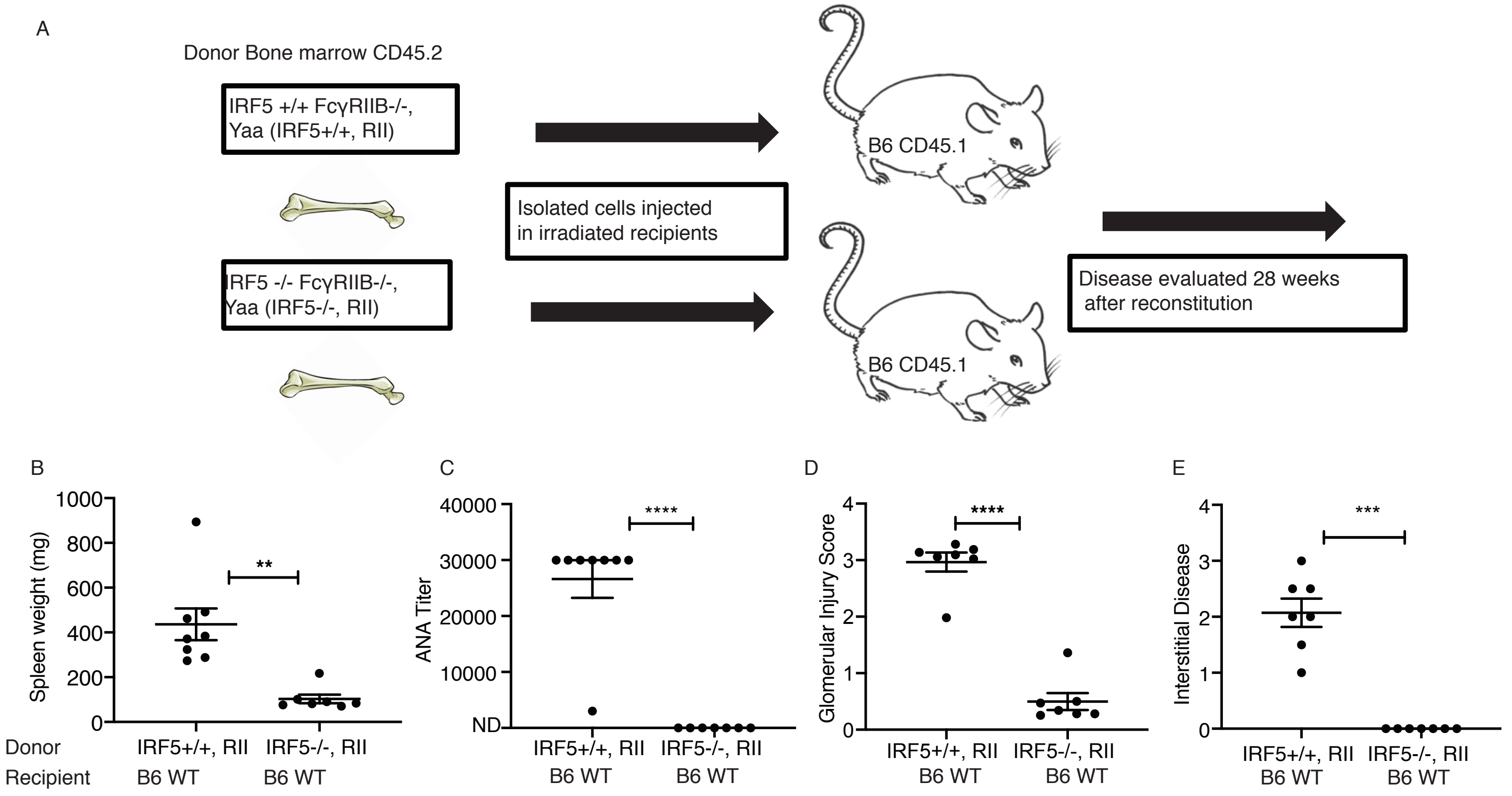

Supplemental Figure 1. IRF5 expression in bone marrow derived cells is sufficient to drive lupus like disease. (A) Schematic of bone marrow chimera generation. (B-E) Analysis of lupus like disease in B6 recipient mice 28 weeks after bone marrow reconstitution with bone marrow from IRF5 sufficient or deficient FcγRIIB–/–Yaa mice. (B) spleen weight, (C) Serum ANA titers, (D and E) Kidney disease as evaluated by glomerular injury score and interstitial disease. Data are means +/- SEM, analyzed using two-tailed, unpaired, Welch's t-test; $^{**}P< 0.01$, $^{***}P < 0.001$, $^{****}P < 0.0001$.

Supplemental Figure 2

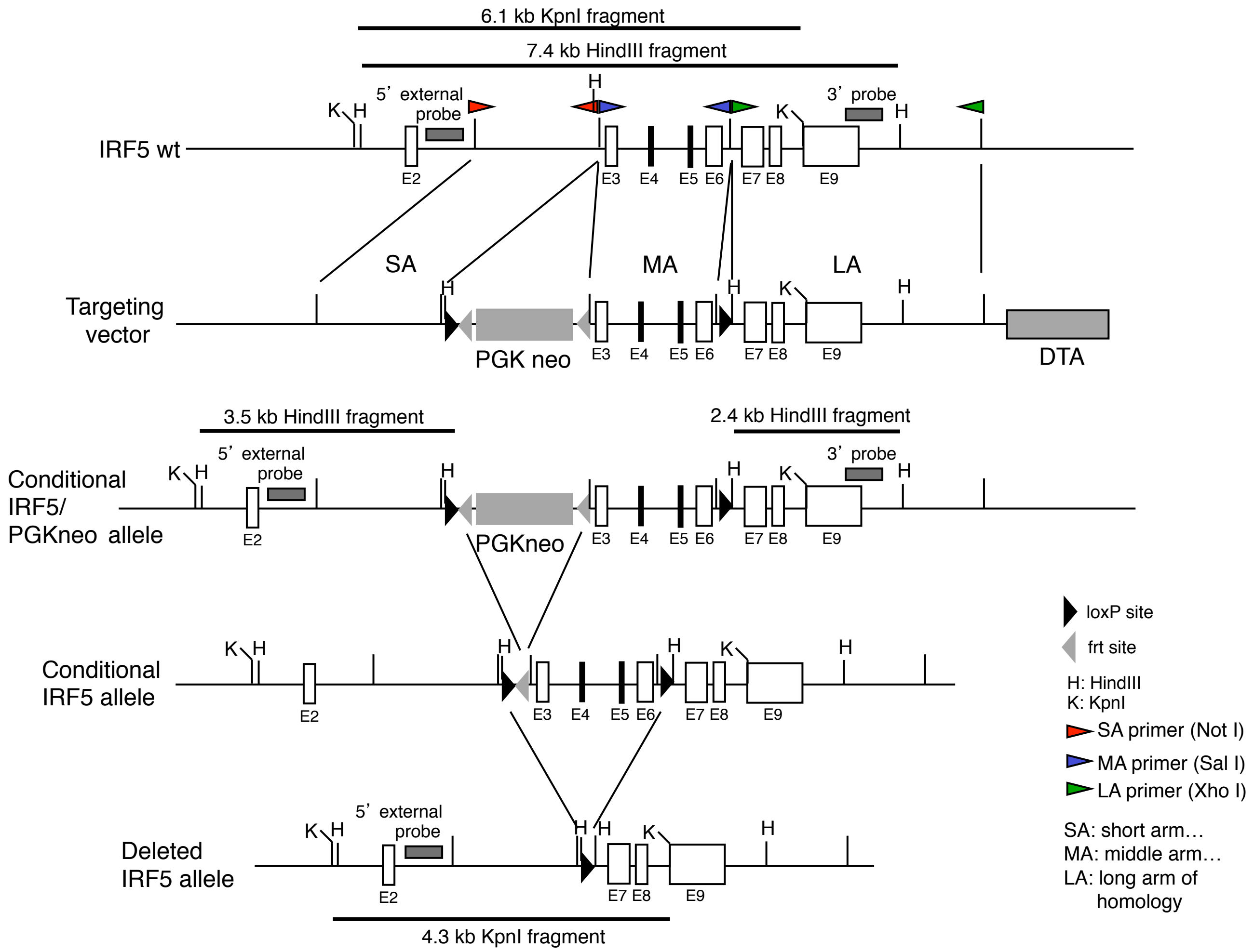

Supplemental Figure 2. Schematic of generation of IRF5 flox allele. Three DNA fragments of the C57BL/6 mice IRF5 locus were inserted into the cloning site of the vector pEZ-Frt-LoxP-DT; 1.6 kb of the region upstream of the IRF5 exon 3, 2 kb of the region between exon 3 to exon 6, and 2.8 kb of the region downstream of exon 6.

Supplemental Figure 3

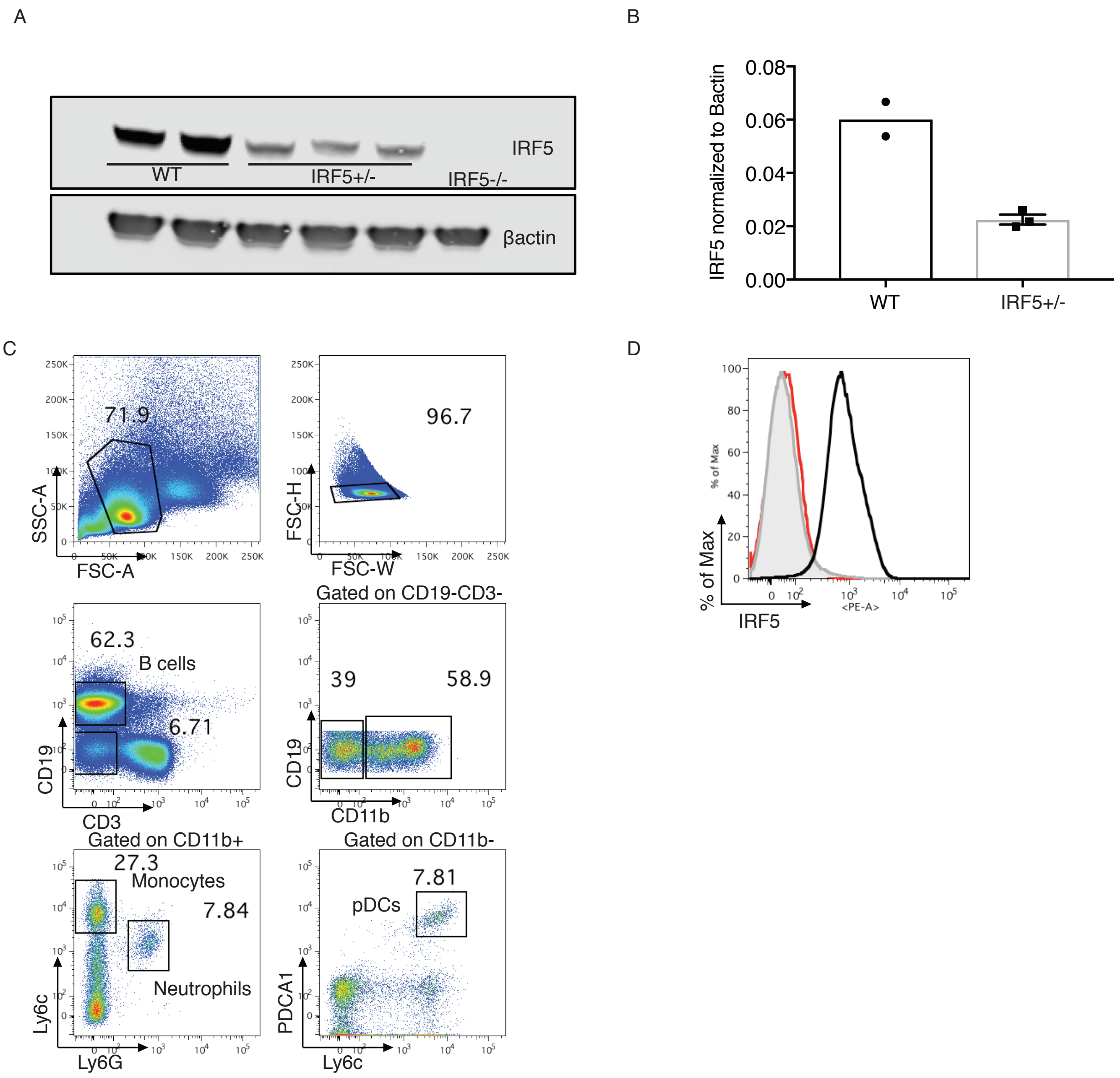

Supplemental Figure 3. Homozygous and heterozygous deletion of IRF5 evaluated by western blot and flow cytometry. (A) Western blot analysis of B cells isolated from 8-week-old FcγRIIB–/–Yaa WT, IRF5 +/- and IRF5-/- mice. (B) IRF5 expression normalized to loading control (actin, lower band in A). Data are means +/-   SEM. (C) Representative example of flow cytometry gating strategy used to identify B cells, monocytes, neutrophils and pDCs. Monocytes were gated as CD19-, CD3-, CD11b+ Ly6C++, Ly6G-. Neutrophils were gated as CD19-,CD3-, CD11b+ Ly6C+, Ly6G+. pDCs were gated as CD19-,CD3-, CD11b-, Ly6C+, PDCA1+. (D) Intracellular flow cytometry for IRF5 gated on CD19+ B cells. Black open histogram represents IRF5 in FcγRIIB–/–Yaa WT B cells, grey tinted histogram represents the isotype control and the red histogram represents IRF5 stain in FcγRIIB–/–Yaa IRF5 -/- B cells.

Supplemental Figure 4

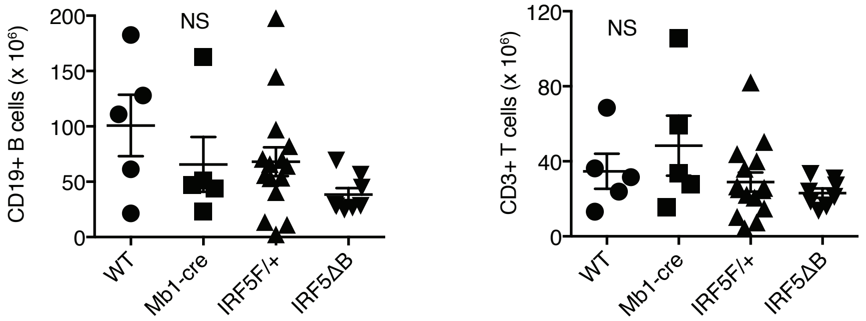

Supplemental Figure 4. Number of splenic CD19+ B cells and CD3+ T cells are similar in FcγRIIB−/−Yaa (WT), mb1cre, IRF5F/+ and IRF5 B mice. Total splenic B cell and T cell numbers in 5 month old WT (n=5), mb1cre (n=5), IRF5F/+ (n=16) and IRF5 B (n=8) mice. Data are means +/- SEM, analyzed using one-way ANOVA with Tukey post hoc test; NS, non-significant.

Supplemental Figure 5

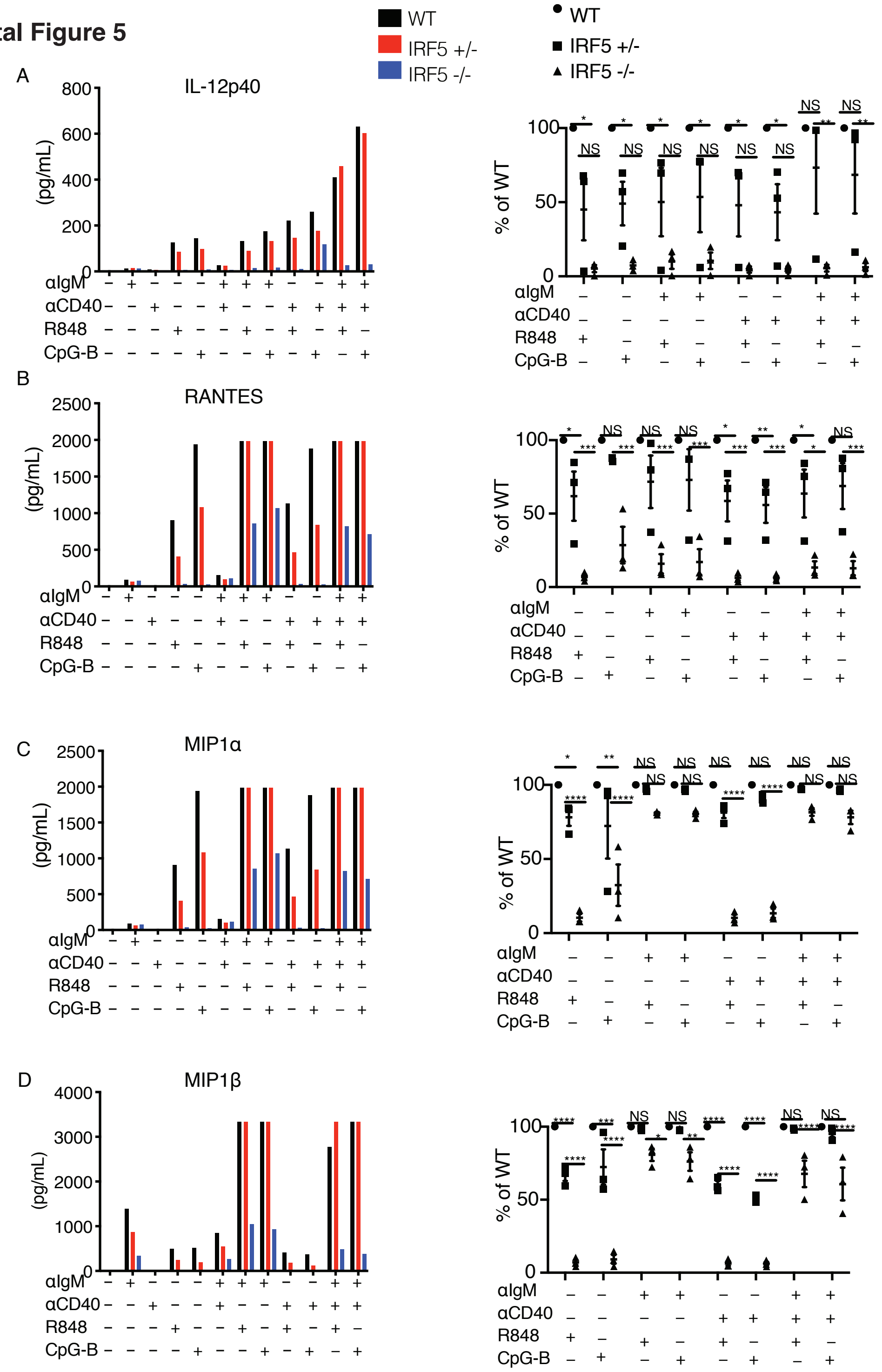

Supplemental Figure 5. The impact of heterozygous and homozygous deletion of IRF5 on B cell derived cytokines and chemokines. B cells were isolated from the spleen of FcγRIIB–/–Yaa IRF5+/+ (WT; black), IRF5 +/- (red) and IRF5 -/- (blue) mice at 8-10 weeks of age and stimulated for 24 hours with anti-IgM, anti-CD40, R848 and CpG-B alone or in combination. Supernatants were analyzed by luminex. Left hand panels are a representative experiment of 3 individual experiments. Right hand panels shows levels of IL-12p40 (A), RANTES (B), MIP-1a(C) and MIP-1b(D) normalized to WT controls (n=3). Data are means +/- SEM, analyzed using two-way ANOVA with Tukey post hoc test; *P < 0.05, **P< 0.01, ***P < 0.001, ****P < 0.0001, NS non-significant.

Supplemental Figure 6

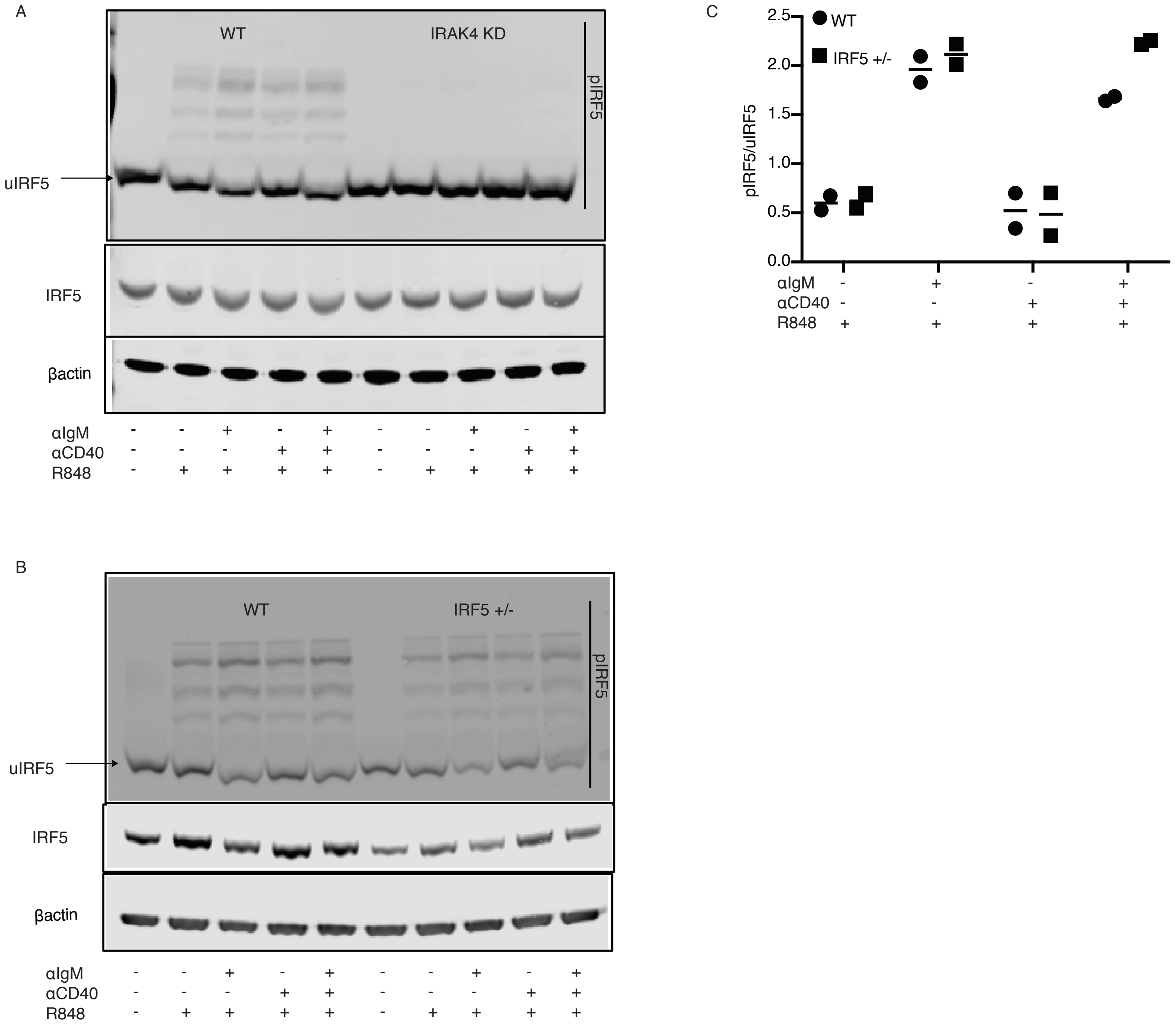

Supplemental Figure 6. IRF5 phosphorylation in B cells from IRF5 +/- and IRAK4 kinase-deficient mice.
(A) B cells were isolated from B6 WT mice or B6 mice deficient in IRAK-4 activity (IRAK-4 KD) and were stimulated with anti-IgM, anti-CD40, and R848 alone or in combination for 2 hours and the protein lysate analyzed using phospho-Tag gel (upper panel) or standard gel (lower panels). A representative example of 2 individual experiments is shown. (B and C) B cells were isolated from the spleens of FcγRIIB−/−Yaa WT and IRF5 +/- mice at 8-10 weeks of age. (B) B cells were stimulated with anti-IgM, anti-CD40, and R848 alone or in combination for 2 hours and the protein lysate analyzed using phospho-Tag gel (upper panel) or standard gel (lower panels). A representative example of 2 individual experiments is shown. (C) Ratio of phosphorylated IRF5 (p-IRF5) to unphosphorylated IRF5 (u-IRF5). p-IRF5 intensity was normalized to the intensity of u-IRF5 (lowest band of IRF5 on p-Tag gel as shown in B).

Supplemental Figure 7

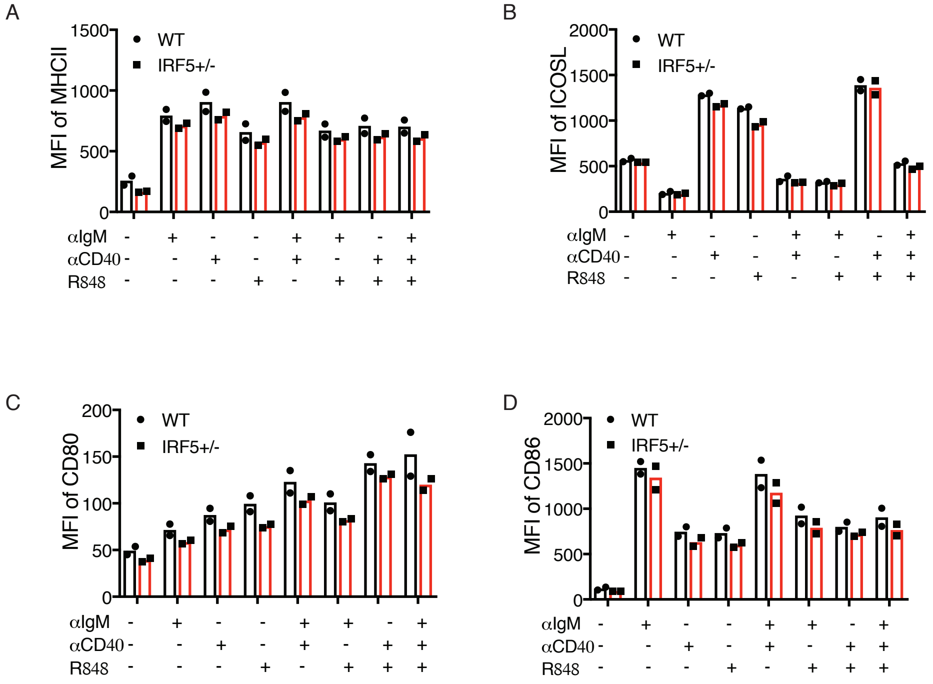

Supplemental Figure 7. Heterozygous deletion of IRF5 does not impact co-stimulatory marker expression on B cells. B cells were isolated from the spleens of FcγRIIB−/−Yaa WT (black) and IRF5 +/- (red) mice at 8-10 weeks of age. The B cells were stimulated with anti-IgM, anti-CD40, and R848 alone or in combination for 24 hours and assessed by flow cytometry for expression of (A) MHC class II, (B) ICOS ligand (ICOSL), (C) CD80 and (D) CD86. A representative experiment (n=2 mice for each genotype) is shown. A total of 4 experiments were performed.
